# Breath volatiles reflect diabetic gastroparesis and its recovery by activating Na_v_1.8-expressing neurons in mice

**DOI:** 10.1038/s12276-026-01824-x

**Published:** 2026-09-24

**Authors:** Seiyoung Hwang, Evonne Kim, Soyun Lee, Daum Lee, Minjoo Park, Ho-Sun Lee, Hyunsoo Chung, Obin Kwon

**Affiliations:** 1 Department of Medical Device Development, Seoul National University College of Medicine, Seoul, South Korea; 2 Department of Biomedical Sciences, BK21 FOUR Biomedical Science Program, Seoul National University College of Medicine, Seoul, South Korea; 3 Department of Internal Medicine and Liver Research Institute, Seoul National University College of Medicine, Seoul, South Korea; 4 Forensic Toxicology Division, Daegu Institute, National Forensic Service, Chilgok, Gyeongsangbukdo South Korea; 5 Genomic Medicine Institute, Medical Research Center, Seoul National University, Seoul, South Korea; 6 Sensory Organ Research Institute, Medical Research Center, Seoul National University, Seoul, South Korea

**Keywords:** Metabolomics, Diabetes complications, Gastroparesis, Therapeutics, Biomarkers

## Abstract

Patients with diabetic gastroparesis (DG) experience chronic, challenging symptoms and currently lack effective treatment options. This study examined whether activating Na_v_1.8-expressing neurons could improve gastric motility and whether volatile organic compounds (VOCs) in exhaled breath could serve as non-invasive indicators of disease status and treatment response. In streptozotocin-induced male diabetic mice, chemogenetic activation of Na_v_1.8-expressing neurons improved gastric motility in DG mice. At the same time, breath VOC profiles differed between DG and non-diabetic controls, with diabetic (3 weeks post-induction) and DG (8–12 weeks) mice showing higher 1-butanol levels than the controls. Two VOCs (2,4-dimethyl-1-heptene and 4-methylheptane) formed a potential treatment-response biomarker panel associated with improved gastric emptying, and the classification model achieved an area under the curve of 0.83 in distinguishing stimulated from non-stimulated states. Our data identified Na_v_1.8-expressing neurons as potential modulators of gastric motility and exhaled VOC patterns as exploratory non-invasive markers of disease dynamics, offering a proof-of-concept framework for neuro-modulatory and breath-test-based strategies to monitor and manage gastroparesis.

## Breath biomarkers reveal insights into diabetic gastroparesis

Diabetic gastroparesis (DG) is a severe complication of diabetes characterized by delayed gastric emptying, leading to symptoms such as nausea and vomiting. This study explores the modulation of Na_v_1.8-expressing neurons to improve gastric motility in a mouse model of DG. Using chemogenetics, researchers activated these neurons, resulting in enhanced gastric emptying, as evidenced by increased plasma acetaminophen levels and improved imaging results. Breath analysis further revealed that VOC profiling holds promise as a non-invasive approach for both disease identification and monitoring treatment response in DG. The study’s findings indicate that targeting Na_v_1.8-expressing neurons could offer a novel therapeutic approach for DG, with breath VOCs serving as non-invasive biomarkers for monitoring treatment efficacy. Future research should explore the applicability of these findings in human models and clarify how modulating Na_v_1.8-expressing neurons improves gastric motility. This summary was initially drafted using artificial intelligence, then revised and fact-checked by the author.

## Introduction

Diabetes mellitus (DM) can cause severe complications that increase mortality rates. Chronic DM complications are classified as either microvascular or macrovascular. Microvascular complications include diabetic retinopathy, nephropathy, and neuropathy. Diabetic neuropathy is one of the most common adverse outcomes of diabetes. It affects the sensory, motor, and autonomic nervous systems, thereby disrupting the reflex and sensory pathways that regulate gastric and intestinal function^1^. Gastrointestinal neuropathy causes various other complications, including gastroparesis, diarrhea, gastroesophageal reflux disease, and impaired accommodation^1,2^.

Among these adverse events, diabetic gastroparesis (DG) is a disease characterized by substantially delayed gastric emptying owing to impaired gastric motility in the absence of mechanical obstruction^3^. DG symptoms include nausea, vomiting, postprandial fullness, and anorexia. If not appropriately treated, it can lead to further morbidities such as severe dehydration and nutritional deficiencies. Although various diagnostic tests are currently used for DG, they have notable constraints. Gastric emptying scintigraphy is widely used in clinical settings, but it cannot be performed for patients in emergencies and requires strict blood glucose control in patients with diabetes. Results from a wireless motility capsule are susceptible to interference from medications that alter gastric acidity. The ^13^C breath test risks yielding false positives and negatives. Radiopaque markers have low diagnostic reliability and often need additional tests. Electrogastrography has an uncertain clinical utility and requires further research^4^. These limitations hinder the accuracy and applicability of these diagnostic modalities.

The management of gastroparesis is also challenging owing to its complex pathophysiology and variable clinical presentation. Current therapies, which include dietary modifications, prokinetic agents, and surgical interventions, offer only limited relief in most instances and are also associated with adverse effects^5^. Recent new endoscopic treatments, such as gastric peroral endoscopic myotomy, have shown promise but require further validation through large-scale studies^6^. Consequently, the prognosis for gastroparesis is generally poor, with many patients experiencing persistent symptoms and complications such as malnutrition and impaired glycemic control^7^. Regenerative medicine is drawing attention as a possible therapeutic option for this disorder, but it is still experimental. Hence, there is a critical need for novel and effective approaches to diagnose and treat delayed gastric emptying.

There has been increasing interests in metabolomics approaches to disease diagnosis. In this context, breath volatilomics, which examines volatile gaseous compounds in the breath, referred to as volatile organic compounds (VOCs), has received considerable attention owing to the abundance of samples and the availability of rapid, non-invasive methods for testing them^8^. Breath VOCs reflect specific biological conditions as they are produced throughout the body and influenced by various factors such as the metabolic status and gut microbiome of an individual^9^. They can also be affected by exogenous factors such as medications, the external environment, and diet. Previous studies have used breath tests to assess gastroparesis^10^, specifically DG^11^. However, these prior reports have primarily focussed on using the non-radioactive carbon isotope ^13^C for diagnosis rather than on analyzing exhaled VOCs. Furthermore, given the limited number of studies on breath VOC analysis in mouse models, we performed VOC analysis of breath samples in the present DG model.

As a potential therapeutic target for gastrointestinal motility disorders, Na_v_1.8 has emerged as a promising candidate. Na_v_1.8 is a voltage-gated sodium channel subtype encoded by the *SCN10A* gene in humans. This channel is expressed in small and medium diameter neurons in the nodose ganglion, dorsal root ganglion, and trigeminal ganglion^12–14^, as well as in ~75% of nodose ganglion neurons^12,15^. Vagal and spinal endings expressing Na_v_1.8 have been distinctly observed in the gastrointestinal mucosa and myenteric plexus^15^. A previous case report has described severe progressive gastroparesis in a female patient with mutations in the *SCN10A* gene^16^. Genetic polymorphisms of *SCN10A* are also reported to be closely associated with functional dyspepsia^17^. Collectively, these findings led us to speculate that modulation of Na_v_1.8-expressing neurons may represent a potential therapeutic approach to control gastrointestinal motility.

This study investigated the modulation of Na_v_1.8-expressing neuronal activity through chemogenetics to alleviate the symptoms of DG. We further identified potential DG biomarkers in exhaled VOCs in the mouse model and evaluated their utility in diagnosis, treatment-response monitoring, and disease classification.

## Materials and methods

### Animals

Adult male wild-type (WT) and genetically engineered C57BL/6J mice were used. WT C57BL/6J mice, Na_v_1.8-cre mice (Jax no. 036564), and ROSA26-LSL-hM3Dq-mCitrine (hM3Dq, Jax no. 026220) mice were obtained from Jackson Laboratory (Bar Harbor, ME, USA). To achieve the chemogenetic activation of Na_v_1.8-expressing neurons using designer receptors exclusively activated by designer drugs (DREADDs), Na_v_1.8-cre mice were crossed with hM3Dq mice (Na_v_1.8^+/^^−^::hM3Dq^+/^^−^; Dq^Nav1.8^). Littermate mice that did not express Na_v_1.8-cre and were therefore not expressing hM3Dq (Na_v_1.8^−/−^::hM3Dq^+/^^−^; Dq^−^) were used as controls for Dq^Nav1.8^. All animals were maintained under controlled conditions at 23 ± 1 °C, 50 ± 10% relative humidity, and a 12:12 h light/dark cycle. Mice were given free access to water and a normal chow diet (Purina, Gyeonggi, Korea).

### Induction of DM

Six-week-old male mice were intraperitoneally injected with streptozotocin (STZ; S0130, Sigma-Aldrich, St Louis, MO, USA) at a dose of 110 mg/kg for 2 consecutive days after a 6-h fasting. The same volume of vehicle (0.1 mol/l sodium citrate buffer, pH 4.5) was injected intraperitoneally into the control mice. The animals were randomly divided into groups at weaning and assigned to drug (STZ or vehicle) treatments. DM induction was assessed by measuring random blood glucose levels from tail-vein blood at 7 days after the last injection; animals with a blood glucose level of 300 mg/dl (16.7 mmol/l) or higher were included in the experiments. Blood glucose levels and body weights were measured once per week until the conclusion of the study.

### AAP absorption test

The acetaminophen (AAP) absorption test was used to evaluate liquid-phase gastric emptying. This method is based on the principle that AAP is poorly absorbed in the stomach and that this absorption occurs in the small intestine. Hence, the plasma concentration of AAP can be used to determine the rate of gastric emptying^18^. This test was conducted in mice at 10 weeks after confirmation of their diabetic status. Mice were fasted for 6 h before the experiment and then orally administered 100 mg/kg of AAP (PHR1005, Sigma-Aldrich). Blood samples were collected from the tail vein into heparinized tubes (no. 365965, Becton, Dickinson, and Company (BD), Franklin Lakes, NJ, USA) at 0, 15, 30, and 60 min after AAP administration. Plasma was then separated by centrifugation at 3000 rpm for 15 min at 4 °C. The plasma samples were stored at −20 °C until analysis.

### Measurement of plasma AAP

Plasma AAP levels were analyzed using a previously described method with some modifications^19^. The working solutions for both AAP and the internal standard (IS, trimipramine-d3) were prepared by diluting their respective stock solutions with HPLC-grade methanol. Briefly, the calibration standards and quality control (QC) samples for AAP were prepared by spiking the mouse plasma with an appropriate volume of the respective working solution. Calibration standards were assessed at a final concentration range of 2−80 μg/ml for AAP. The QC samples were prepared at a concentration of 0.2 μg/ml and 20 μg/ml in five replicates. An aliquot of 5 μl of plasma sample containing AAP was mixed with 10 μl of IS working solution in a 2 ml Eppendorf tube. An aliquot of 10 μl of IS was added to each sample with 35 μl of acetonitrile–methanol (90:10, v/v), yielding a final IS concentration of 0.5 μg/ml. After 10 min of agitation at room temperature followed by centrifugation at 3000×*g* for 5 min and passing through a centrifugal filter (0.22 μm filter unit; EMD Millipore, Billerica, MA, USA), the supernatant was injected into the chromatographic system.

Liquid chromatography–tandem mass spectrometry (LC–MS/MS) analysis was performed using an AB SCIEX Qtrap 4500 system (AB Sciex, Markham, Canada) consisting of an Agilent 1290 infinity ultrahigh-performance liquid chromatography system (Agilent Technologies, Santa Clara, CA, USA) using a Kinetex XB-C18 (50 × 3.0 mm, 2.6 μm, particle size 100 Å) column (Phenomenex, Torrance, CA, USA)^19,20^. Chromatographic separation was performed at 40 °C with a binary gradient consisting of 0.2% formic acid and 2 mM ammonium formate in water (mobile phase A) and 0.2% formic acid and 2 mM ammonium formate in acetonitrile (mobile phase B). The gradient profile was as follows: 2% B in 0.00–1.00 min, 100% B in 12.00–15.00 min, and 2% B in 15.10–17.50 min. The flow rate was a constant 0.5 ml/min, and the injection volume was 1 μl. Electrospray ionization–MS/MS detection was performed in the multiple reaction monitoring (MRM) mode, with two transitions for AAP and two for IS in positive ionization mode. The MRM parameters of AAP and IS are listed in Table 1. Compound-independent MS parameters were as follows: curtain gas, 10 psi; nebulizer gas (GS1), 55 psi; drying gas (GS2), 55 psi; ionization voltage, 5500 V; ion source temperature, 500 °C. Collision-induced decay was performed using nitrogen gas (gas flow set to medium). The peak area of the first transition was used for quantification. Instrument control and data processing were performed using Analyst software version 1.6.2 from AB SCIEX.

**Table 1:** Multiple reaction monitoring (MRM) conditions for acetaminophen (AAP) and the trimipramine-d_3_ internal standard.

Targets	Q1^a^	Q3^b^	RT (min)	DP (V)	EP (V)	CE (V)	CXP (V)
AAP (mw 151.16, C_8_H_9_NO_2_)	152	110.1	1.4	70	4.5	35	4
		65	1.4	70	10	41	4
Trimipramine-d_3_ (mw 297.46, C_20_H_23_D_3_N_2_)	298.3	103	6.6	81	3	23	8
		193.3	6.6	81	3	51	8

^a^Precursor ion (*m*/*z*).

^b^Product ion (*m*/*z*). CE, collision energy; CXP, collision cell exit potential; DP, declustering potential; EP, entrance potential; mw, molecular weight; RT, retention time.

Accuracy was determined using the relative error (−2.0 and −1.3 for 0.2 μg/ml and 20 μg/ml of AAP) and precision via the relative standard deviation (4.7 and 3.9 for 0.2 μg/ml and 20 μg/ml of AAP). The recovery was calculated by the ratio of the peak area obtained from the analyte spiked into a pre-extraction matrix to that obtained from the substance spiked into the post-extraction matrix. The matrix effect was assessed by the ratio of the analyte peak areas in the post-extracted matrix solution to those in the aqueous solution. The matrix effect and recoveries were in the range of 90–105% for all analytes. The limit of detection was defined as the concentration at which a signal-to-noise ratio of 3:1 was achieved.

### Western blotting

Frozen mouse stomach samples were homogenized in a lysis solution containing RIPA lysis and extraction buffer (no. 89900, Thermo Fisher Scientific, Waltham, MA, USA), phosphatase inhibitor cocktail (PhosSTOP™; no. 4906845001, Roche, Basel, Switzerland), and protease inhibitor cocktail (no. 04693159001, Roche). Following centrifugation of the lysates, the protein concentration in the supernatant was determined using a BCA Protein Assay Kit (no. 23228, Thermo Fisher Scientific). Protein samples were then resolved on 8% SDS–polyacrylamide gel and transferred to a polyvinylidene fluoride membrane (IPVH00010, Millipore). After blocking for 1 h at room temperature with 5% skimmed milk in 1× TBS containing 0.1% Tween 20, the membranes were incubated overnight at 4 °C with the following primary antibodies: rabbit monoclonal anti-c-Kit (no. 3074S, Cell Signaling, Danvers, MA, USA), mouse monoclonal anti-HO-1 (ab13248, Abcam, Cambridge, UK), and mouse monoclonal anti-β-actin (SC-81178, Santa Cruz, Dallas, TX, USA). After several washes on the following day, horseradish peroxidase-conjugated secondary antibodies were applied for 1 h at room temperature. Signals were subsequently visualized using an enhanced chemiluminescence substrate (no. 32106, Thermo Fisher Scientific) and detected on an Amersham Imager 600 (GE Healthcare, Chicago, IL, USA). ImageJ software (NIH, Bethesda, MD, USA) was used to quantify and analyze the signal intensity.

### Breath VOC sampling

The breath VOC sampling setup is depicted in Fig. 2a. The sampling device consisted of a mouse stabilizer and a polytetrafluoroethylene (PTFE) bag (Scentroid, Toronto, ON, Canada). Each animal was placed in a stabilizer consisting of a tip-cut 50 ml Falcon tube and a PTFE disc for support. This disc ensured that the mouse remained in place and prevented the escape of breath. PTFE bags were pre-cleaned with clean air and heated at 60 °C for 1 h. A PTFE bag filled with 1 l of VOC-free synthetic air (20% O_2_ and 80% N_2_) was then connected to the mouse stabilizer. After 30 min of breath collection in this device, a mouse was released, and the PTFE bag containing the collected breath was sealed by clipping. A total of 750 ml of collected breath in the PTFE bag was concentrated using a sampling pump (GilAirplus, Sensidyne, St Petersburg, FL, USA) at a flow rate of 200 ml/min onto a Bio-monitoring adsorbent tube (Markes International Ltd, Llantrisant, UK). VOC-adsorbed tubes were sealed and immediately stored at –20 °C to prevent sample loss.

### Gas chromatography–mass spectrometry (GC–MS)

VOC identification was carried out using a thermal desorption Unity-xr (Markes International Ltd, Llantrisant, UK) coupled with a gas chromatography GC-Agilent 8890 and a mass spectrometer MS-Agilent 5977 B (Agilent Technologies). Briefly, the VOCs trapped in the desorption tubes were released at 300 °C under a helium flow using thermal desorption. The sample for analysis was then transmitted to a cold trap programmed at 20 °C for sample concentration. Subsequently, the sample was injected into the GC column (Agilent, column: DB -5 MS UI, 60 m × 0.25 mm ID, coated with 0.25 μm). Compounds were separated using a temperature gradient initiated at 40 °C for 5 min, followed by an increase of 7 °C/min until 280 °C was reached. This final temperature was maintained for 3 min. All VOCs were then identified utilizing Mass Hunter Quantitative, Mass Hunter Unknown Analysis, and the NIST 17 mass spectral library. NIST 17 was uploaded to Unknown Analysis; sample chromatograms were deconvoluted, and all features were identified. A compound was identified only if the retention time was within 20 min, the mass spectra matched, and the coefficient of agreement exceeded 80. For selected compounds, identification was further validated using authentic standards by injecting 1 μl of 1 μg/μl solutions into sorbent tubes under a nitrogen flow (40 ml/min) with a purge time of 2 min, followed by a comparison of mass spectra using the NIST MS Search function within the Agilent MassHunter Qualitative Analysis software (Agilent Technologies).

### Drug administration

To stimulate Na_v_1.8-expressing neurons, male Dq^Nav1.8^ mice and their Dq^−^ littermates were administered with clozapine *N*-oxide (CNO; HB6149, HelloBio, Bristol, UK; dissolved in 0.9% sterile saline at 1 mg/ml) via intraperitoneal injection. For the AAP absorption test, mice received CNO at 10 mg/kg intraperitoneally 30 min before the intragastric gavage of the AAP solution. For in vivo fluorescence imaging, the mice received CNO at 3 mg/kg intraperitoneally 25 min before intragastric gavage of IVISense GastroIntestinal 750 (NEV11121, Revvity, Waltham, MA, USA). For breath VOCs and tissue sampling, mice were intraperitoneally administered CNO at 1 mg/kg 30 min before VOC sampling. All drugs were administered to each mouse in a randomized order.

### In vivo fluorescence imaging

In vivo fluorescence imaging was conducted using the IVIS Spectrum system (Revvity) following intragastric gavage of IVISense GastroIntestinal 750 in phosphate-buffered saline (0.25 nmol/100 μl/mouse). The mice were fasted overnight to allow for stomach emptying. Fluorescence data were acquired by targeting the gastric region immediately before and 5, 15, and 60 min after intragastric gavage. Fluorescence images were analyzed using Living Image software (version 4.7.4, Revvity). The higher gastric fluorescence signal measured at 5 min or 15 min after gavage was used as the reference value, and the percentage of gastric emptying was calculated as [{1 − (gastric fluorescence signal at 60 min/reference gastric fluorescence signal)} * 100] and normalized to the body weight of each mouse. Any data points that deviated more than two standard deviations from the mean of the dataset were considered outliers and excluded from further analysis.

### Statistical analysis and data pre-processing

Unpaired *t* tests were used to compare body weights, random blood glucose levels, areas under the curve (AUC) for plasma AAP concentrations, gastric emptying ratios, VOC concentrations, and the number of c-fos-positive cells between the animal groups. Two-way repeated-measures analysis of variance (ANOVA) with a Bonferroni, Fisher’s least significant difference (LSD), or Sidak’s post hoc test was used to compare plasma AAP concentrations over various time points or to compare 1-butanol concentrations. One-way ANOVA with an LSD post hoc test was used to compare the AUCs of plasma AAP concentrations and the gastric emptying ratios among three or more groups. All analyses were conducted using SPSS Statistics version 29 (IBM Corp., Armonk, NY, USA).

### Statistical processing of breath VOCs

To analyze breath VOCs, GC–MS peaks were tentatively matched to compounds using the NIST 17 library, with a match factor ≥ 80% and retention time < 20 min. During pre-processing, peaks formed by GC–MS analysis were grouped across samples by *m*/*z* value (tolerance: 0.001) and retention time (tolerance: 0.1 s). VOCs with more than 50% of missing values were removed, and the remaining missing values were imputed with one-fifth of the minimum positive value of each variable. Data were filtered based on the interquartile range to exclude non-informative variables. Log transformation (base 10) was applied for normalization and auto-scaling. To detect outliers, a random forest algorithm was applied. Pre-processing and statistical analysis were performed with MetaboAnalyst 6.0 (https://www.metaboanalyst.ca/)^21^.

After pre-processing, fold change analysis and Wilcoxon rank-sum tests were performed to identify statistically significant VOCs (|log_2_ fold change (FC)| ≥ 1, *P* < 0.05). A volcano plot was generated to visualize differences between the two groups with log_2_ fold change on the *x*-axis and −log_10_(*P*-value) on the *y*-axis. Clustering analysis, such as partial least squares discriminant analysis (PLS-DA), was used for multivariate statistical analysis. Variable importance in projection (VIP) scores for features utilized to form PLS-DA plots were also calculated. Enrichment analysis was conducted to define over-represented subgroups of chemical structures. Univariate and multivariate receiver operating characteristic (ROC) analyses were performed using a Random Forest algorithm in MetaboAnalyst to evaluate the diagnostic performance of the VOCs and their potential as disease biomarkers, particularly those with VIP scores greater than 1.0. For enrichment analysis, VOC compound names were converted to PubChem Compound Identification (CID), and over-represented chemical subgroups were identified using MetaboAnalyst based on chemical structure classification. A combinational model to distinguish between two groups was constructed using selected VOCs, and its robustness was evaluated using ROC analysis and permutation testing.

### Blinding

Experimental animal group allocations and drug administration could not be blinded; however, subsequent procedures, including data processing and analysis, were conducted in a blinded manner, with the experimenter unaware of each animal’s group assignment.

## Results

### Establishment of a diabetic gastroparesis mouse model

**Fig. 1:**
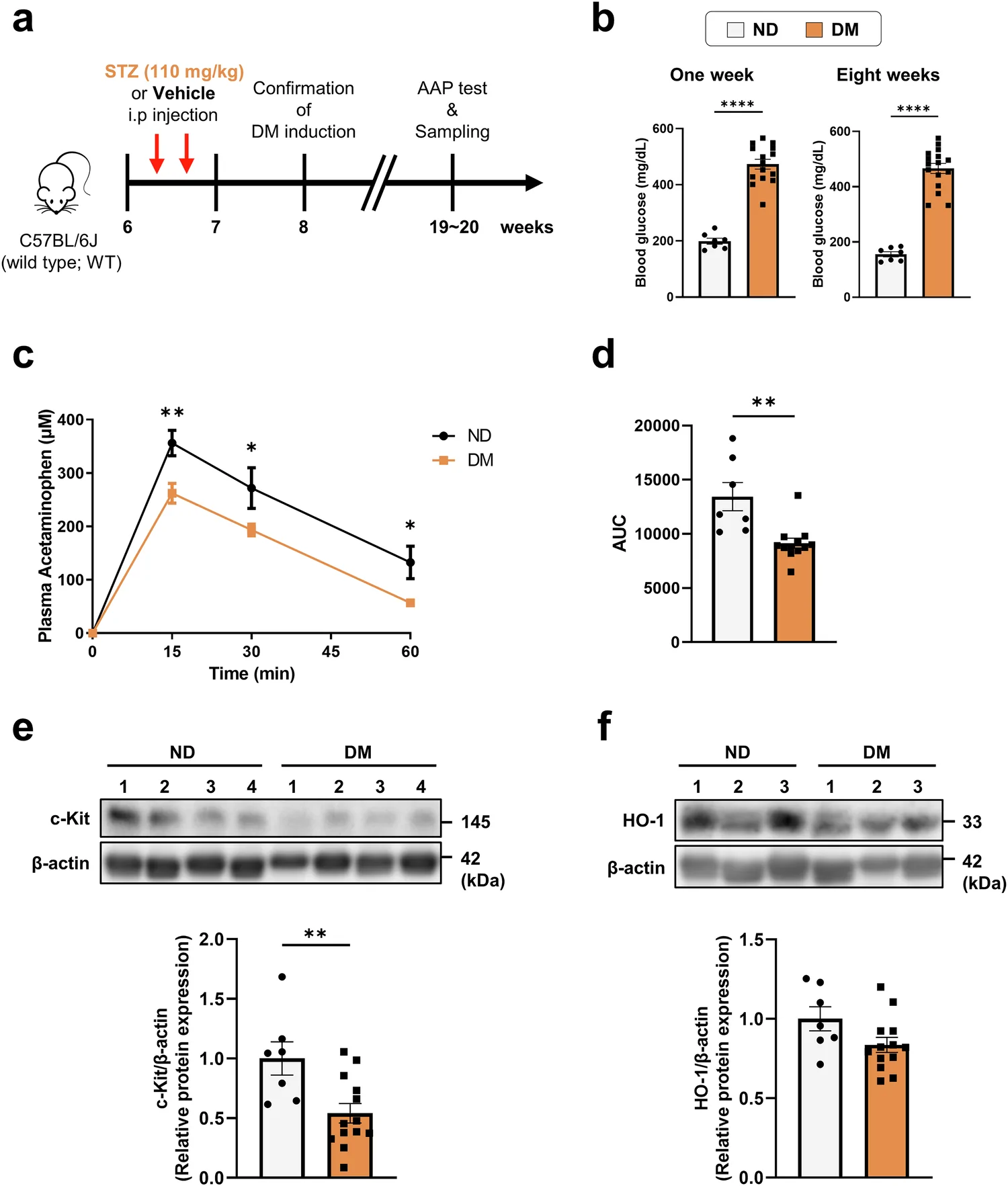
Establishment of a streptozotocin (STZ)-induced diabetes mellitus (DM) and diabetic gastroparesis (DG) mouse model. **a**, Experimental scheme. **b**, Blood glucose levels at 1 and 8 weeks after STZ injection. **c**, Liquid-phase gastric emptying was assessed in mice fasted for 6 h using the acetaminophen (AAP) absorption test. **d**, AAP plasma concentration and associated area under the curve (AUC) values. Western blotting and quantification of relative expression of c-Kit (part **e**) and HO-1 proteins (part **f**) in the stomach. Statistical analysis was performed using an unpaired *t* test or two-way repeated-measures analysis of variance with Bonferroni post hoc. Data are presented as mean ± SEM. ND-WT, *n* = 7; DM-WT, *n* = 15. ^*^P < 0.05; ^**^P < 0.01; ^****^P < 0.0001. ND, non-diabetic; i.p., intraperitoneal; SEM, standard error of the mean.

An STZ-induced DM mouse model was used in this study (Fig. 1a). These mice (DM-WT) showed a significant increase in random blood glucose levels compared with non-diabetic wild-type (ND-WT) controls. This difference was maintained throughout the experimental period (Fig. 1b). To evaluate liquid-phase gastric emptying, the AAP absorption test was performed 11 weeks after DM induction^18^. The plasma AAP levels at every time point (15, 30, and 60 min) were significantly decreased in the DM-WT mice (Fig. 1c,d), indicating delayed gastric emptying. Compared with ND-WT mice, DM-WT mice showed significantly lower expression levels of the c-Kit protein in the stomach (Fig. 1e), suggesting loss of interstitial cells of Cajal (ICC), a common finding in DG^22^. Previous studies reported that heme oxygenase-1 (HO-1) expression was upregulated in the early stages of diabetes as an endogenous defense and cytoprotective mechanism but subsequently declined to levels comparable to those of non-diabetic controls with the development of delayed gastric emptying^23^. Consistent with this, our data demonstrated that HO-1 protein expression was significantly elevated in the early stage of diabetes (Supplementary Fig. 1). By contrast, no significant difference in HO-1 expression was observed at the time point when delayed gastric emptying developed (Fig. 1f). Importantly, additional analyses indicated that the delayed gastric emptying phenotype was not attributable to age-related maturation (Supplementary Fig. 2a,b) and was not present at the early diabetic stage (3 weeks after DM induction) (Supplementary Fig. 2c,d), suggesting that gastric emptying delay emerged in association with prolonged diabetes. These findings confirmed the successful establishment of a DG mouse model.

### Distinct breath VOCs in diabetic gastroparesis

**Fig. 2:**
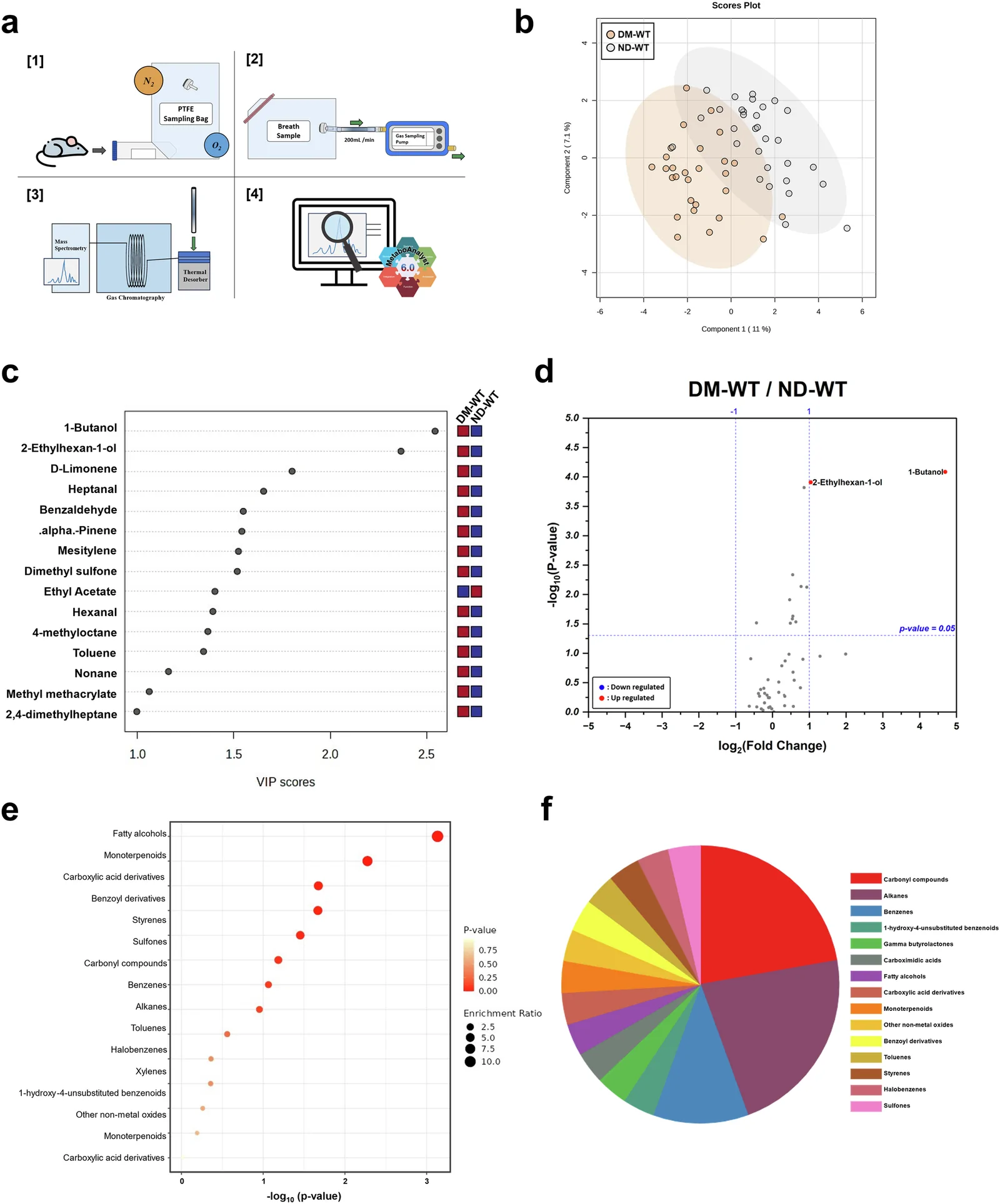
Identification of potential breath volatile organic compound (VOC) biomarkers through statistical screening of compounds detected in DM-WT and ND-WT mice at 12 weeks after diabetes induction. **a**, Schema for breath VOC sampling and analysis: [1] collection of mouse breath in a PTFE bag; [2] absorption of VOCs into a sorbent tube; [3] sample injection and GC–MS analysis; and [4] peak and statistical analysis. **b**, Partial least squares discriminant analysis (PLS-DA) plot showing breath VOC grouping patterns in DM-WT and ND-WT mice. **c**, Variable importance in projection (VIP) scores for breath VOCs contributing to the PLS-DA plot. The colors of the boxes on the right indicate higher (red) or lower (blue) intensity of each VOC within each group. **d**, Volcano plot of breath VOCs comparing DM-WT and ND-WT, with differential VOCs identified using the Wilcoxon rank-sum test (|log_2_FC|≥ 1.0, *P* < 0.05). **e**, Bubble plot showing enrichment analysis of breath VOC subclasses for DM-WT and ND-WT mice, based on their chemical structures. **f**, Pie chart showing VOCs detected in DM-WT and ND-WT mice. DM, diabetes mellitus; FC, fold change; GC–MS, gas chromatography–mass spectrometry; PTFE, polytetrafluoroethylene; WT, wild-type.

To examine breath VOC patterns in each animal group, breath samples were collected from ND-WT and DM-WT mice at 12 weeks after DM induction. The VOCs in concentrated breath samples were measured by GC–MS (Fig. 2a). All detected VOCs in ND-WT and DM-WT mice were displayed as a hierarchical clustering heatmap (Supplementary Fig. 3). PLS-DA plots were then generated to visualize grouping patterns of the breath VOCs between the two groups (Fig. 2b). Along with the PLS-DA plot, VIP scores were provided for the features that discriminated DM-WT mice from ND-WT mice. Interestingly, almost all features were elevated in DM-WT mice, most notably 1-butanol and 2-ethylhexan-1-ol (Fig. 2c). In a subsequent volcano plot, 1-butanol and 2-ethylhexan-1-ol were identified as statistically significant compounds differentiating DM-WT and ND-WT mice, with both compounds showing higher concentrations in the DM-WT samples (|log_2_FC| ≥ 1.0, *P* < 0.05) (Fig. 2d). To assess breath VOCs during the early phase of DM, before the onset of DG, we also analyzed samples from ND-WT and DM-WT mice at 3 weeks after DM induction (Supplementary Fig. 4). VOCs that might have resulted from other diabetic complications were inferred by removing the results from the analysis of ND-WT mice from those obtained in the comparative analyses at 3 and 12 weeks after DM induction (Supplementary Fig. 5). DM-WT mice showed higher levels of 1-butanol at both 3 and 12 weeks after DM induction (Fig. 2d and Supplementary Fig. 4c). As the mice aged, 1-butanol levels declined markedly in the ND-WT mice. Still, they persisted at elevated levels in the DM-WT mice (Supplementary Fig. 6), resulting in a more pronounced distinction following DG onset (Fig. 2d). These findings suggest that 1-butanol in the breath is associated with diabetes and with DG that can develop in the later stages of diabetes. Enrichment analysis indicated that fatty alcohols, monoterpenoids, carboxylic acids, and benzoyl derivatives comprised the largest proportions of detected breath VOCs (Fig. 2e). A subsequent analysis presented as a pie chart revealed that the majority of the detected VOCs were alkanes and carbonyl compounds (Fig. 2f).

### Improvement in gastric motility following activation of Na_v_1.8-expressing neurons

**Fig. 3:**
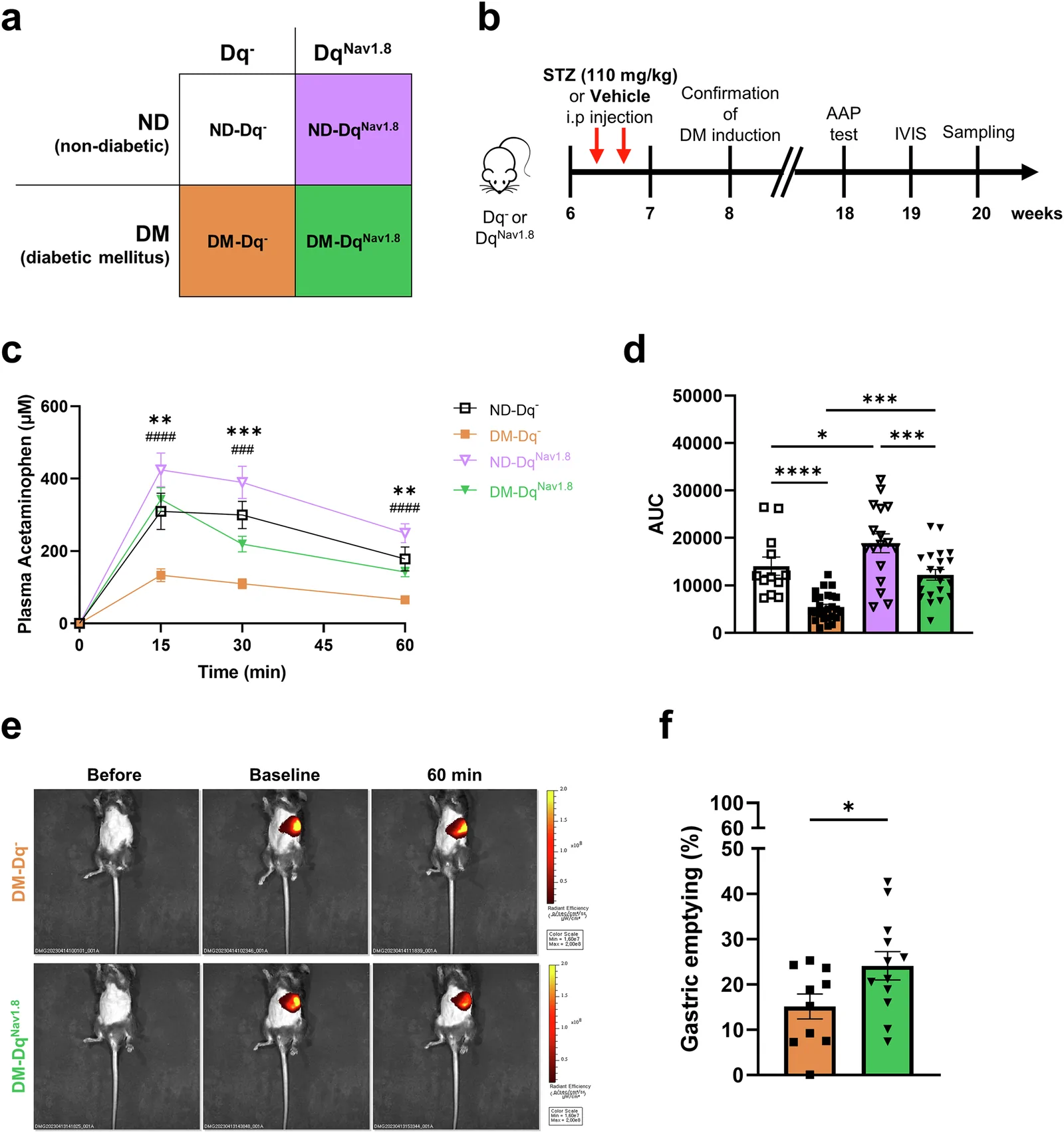
Improvement of gastric emptying by activation of Na_v_1.8-expressing neurons. **a**, Quadrant description of the four experimental animal groups. **b**, Experimental scheme. **c**,**d**, Acetaminophen (AAP) absorption test was conducted in all groups. **c**, Serial plasma concentrations of AAP. ^**^P < 0.01 and ^***^P < 0.001 for ND-Dq^−^ versus DM-Dq^−^; ^###^*P* < 0.001 and ^####^*P* < 0.0001 for DM-Dq^−^ versus DM-Dq^Nav1.8^. **d**, Area under the curve (AUC) values for AAP concentration in part **c**. ND-Dq^−^, *n* = 12; DM-Dq^−^, *n* = 26; ND-Dq^Nav1.8^, *n* = 17; DM-Dq^Nav1.8^, *n* = 21. **e**,**f**, Gastric emptying test conducted in DM-Dq^−^ and DM-Dq^Nav1.8^ mice using the IVIS Spectrum. Representative images (part **e**) and gastric emptying ratio (%) (part **f**). DM-Dq^−^, *n* = 10; DM-Dq^Nav1.8^, *n* = 12. ^*^P < 0.05; ^***^P < 0.001; ^****^P < 0.0001. All groups received clozapine *N*-oxide (CNO) 30 min before intragastric gavage of the AAP solution (parts **c** and **d**) or 25 min before intragastric gavage of the fluorescence probe (parts **e** and **f**) for their chemogenetic activation. Statistical analysis was performed using two-way repeated-measures analysis of variance (ANOVA) in part **c**, one-way ANOVA with least significant difference (LSD) post hoc test in part **d**, or unpaired *t* test in part **f**. Data are presented as mean ± SEM. SEM, standard error of the mean. DM, diabetes mellitus.

To investigate the effects of modulating Na_v_1.8-expressing neurons on gastric motility in the DG mouse model, Na_v_1.8-specific Gq-DREADD mice (Dq^Nav1.8^ mice) and their controls (Dq^−^ mice) were generated. The mice were divided into four groups based on the presence of diabetes and the ability to activate Na_v_1.8-expressing neurons (Fig. 3a). Gastric emptying rates were assessed using both the AAP absorption test and IVIS imaging following DG induction (Fig. 3b). Chemogenetic activation of Na_v_1.8-expressing neurons by CNO injection was confirmed by the increased number of c-Fos-positive cells in the lateral septal nucleus, bed nucleus of the stria terminalis, amygdala, and paraventricular nucleus of the hypothalamus^24–26^ (Supplementary Fig. 7). In both the Dq^−^ and Dq^Nav1.8^ groups, DM mice exhibited a lower plasma AAP concentration compared with ND mice (Fig. 3c,d), consistent with delayed gastric emptying as a consequence of diabetes. Notably, DM-Dq^Nav1.8^ mice had significantly higher plasma AAP levels relative to DM-Dq^−^ mice, reaching levels comparable to those of ND-Dq^−^ mice (Fig. 3c,d). This result indicates that the activation of Na_v_1.8-expressing neurons ameliorates diabetes-induced gastric dysmotility. A similar effect was observed in the ND group, as ND-Dq^Nav1.8^ mice had higher plasma AAP concentrations than ND-Dq^−^ mice (Fig. 3c,d). To further corroborate these findings, gastric emptying was visualized using the IVIS Spectrum system. Consistent with the plasma AAP concentration data, DM-Dq^Nav1.8^ mice showed a significantly higher gastric emptying rate compared with DM-Dq^−^ mice (Fig. 3e,f). Collectively, these results suggested that stimulation of Na_v_1.8-expressing neurons enhances gastric emptying and may mitigate the manifestations of DG.

### Activation of Na_v_1.8-expressing neurons to accelerate gastric emptying and its impact on breath VOCs

**Fig. 4:**
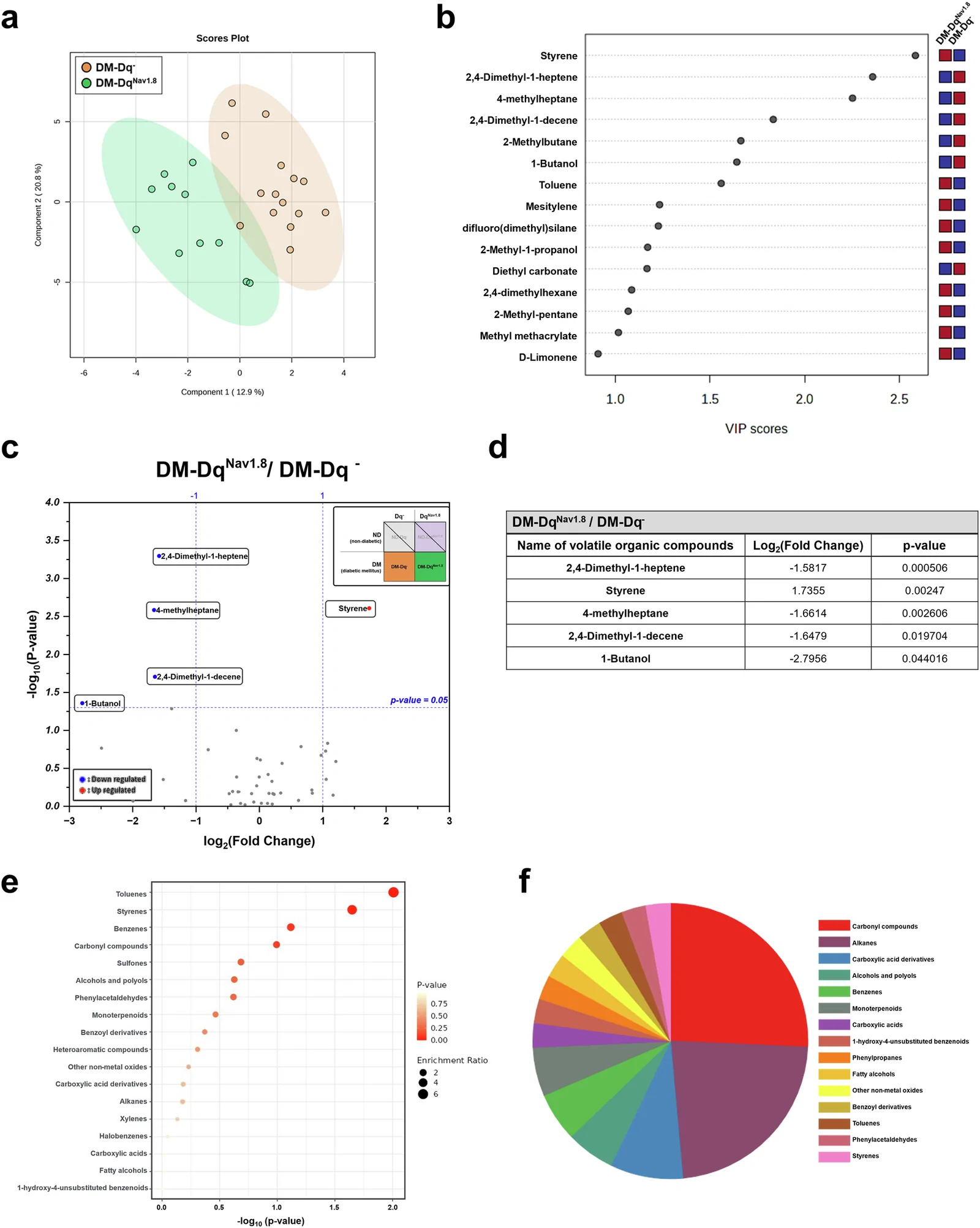
Potential breath VOC biomarkers for improved gastric emptying via activation of Na_v_1.8-expressing neurons. **a**, Partial least squares discriminant analysis (PLS-DA) plot visualizing the grouping patterns of breath volatile organic compound (VOC) concentrations in DM-Dq^Nav1.8^ and DM-Dq^−^ mice. **b**, Variable importance in projection (VIP) scores for breath VOCs contributing to the PLS-DA plot. The colors of the boxes on the right indicate higher (red) or lower (blue) intensity of each VOC within each group. **c**, Volcano plot of breath VOCs comparing DM-Dq^Nav1.8^ and DM-Dq^−^ mice (DM-Dq^Nav1.8^/DM-Dq^−^), with differential VOCs identified using the Wilcoxon rank-sum test (|log_2_FC|≥ 1.0, *P* < 0.05). Rectangular boxes around VOC names indicate commonly detected VOCs following activation of Na_v_1.8-expressing neurons in both part **c** and Supplementary Fig. 9d. **d**, Summary of breath VOCs identified as statistically significant with greater than twofold differences (|log_2_FC|≥ 1.0, *P* < 0.05). **e**, Bubble plot showing enrichment analysis of breath VOCs from DM-Dq^Nav1.8^ and DM-Dq^−^ mice, based on chemical subclass structures. **f**, Pie chart of VOCs detected in DM-Dq^Nav1.8^ and DM-Dq^−^ mice. All groups received CNO 30 min before exhaled-breath sampling for chemogenetic activation. CNO, clozapine *N*-oxide; DM, diabetes mellitus; FC, fold change.

To examine breath VOCs related to gastric motility, particularly in the DG mice, breath VOCs in both DM groups (DM-Dq^Nav1.8^ and DM-Dq^−^) were analyzed at 30 min after CNO injection, with the ND groups (ND-Dq^Nav1.8^ and ND-Dq^−^) serving as controls. The VOC profiles in each group are shown as heatmaps (Supplementary Figs. 8 and9a). To identify VOC grouping patterns within each group of mice, PLS-DA plots were generated, followed by VIP score plots showing the top 15 features contributing to the PLS-DA models (Fig. 4a,b and Supplementary Fig. 9b,c). Regardless of the presence or absence of diabetes, styrene was found to be enriched, and four other substances (2,4-dimethyl-1-heptene, 4-methylheptane, 1-butanol, and 2,4-dimethyl-1-decene) were detected at lower levels in both Dq^Nav1.8^ groups (ND-Dq^Nav1.8^ and DM-Dq^Nav1.8^), following the activation of Na_v_1.8-expressing neurons (Fig. 4c,d and Supplementary Fig. 9d,e). It was speculated that this reduction was associated with enhanced gastric motility (Fig. 3). Notably, 1-butanol, which was the most prominent compound that could discriminate DM-WT from ND-WT (Fig. 2d), was significantly decreased after the activation of Na_v_1.8-expressing neurons (Fig. 4c,d and Supplementary Fig. 9d,e). DM-Dq^Nav1.8^ mice exhibited significantly lower 1-butanol concentrations than DM-Dq^−^ mice (Fig. 4c,d and Supplementary Fig. 10a), and a similar reduction was observed in ND-Dq^Nav1.8^ mice compared with ND-Dq^−^ mice (Supplementary Figs. 9d,e and 10b). There were no changes in the VOC levels caused by the CNO drug itself (Supplementary Fig. 10c). These results suggest the potential clinical utility of various breath VOCs, especially 1-butanol, as indicators of neuronal activation and gastric motility. The enrichment analysis revealed that toluenes, styrenes, benzenes, and carbonyl compounds constituted the majority of statistically significant breath VOCs, as indicated by their high enrichment ratios and low *P*-values (Fig. 4e). A subsequent pie chart further revealed that alkanes and carbonyl compounds represented the major portion of these breath VOCs (Fig. 4f).

### Potential classification models for monitoring diabetic gastroparesis

**Fig. 5:**
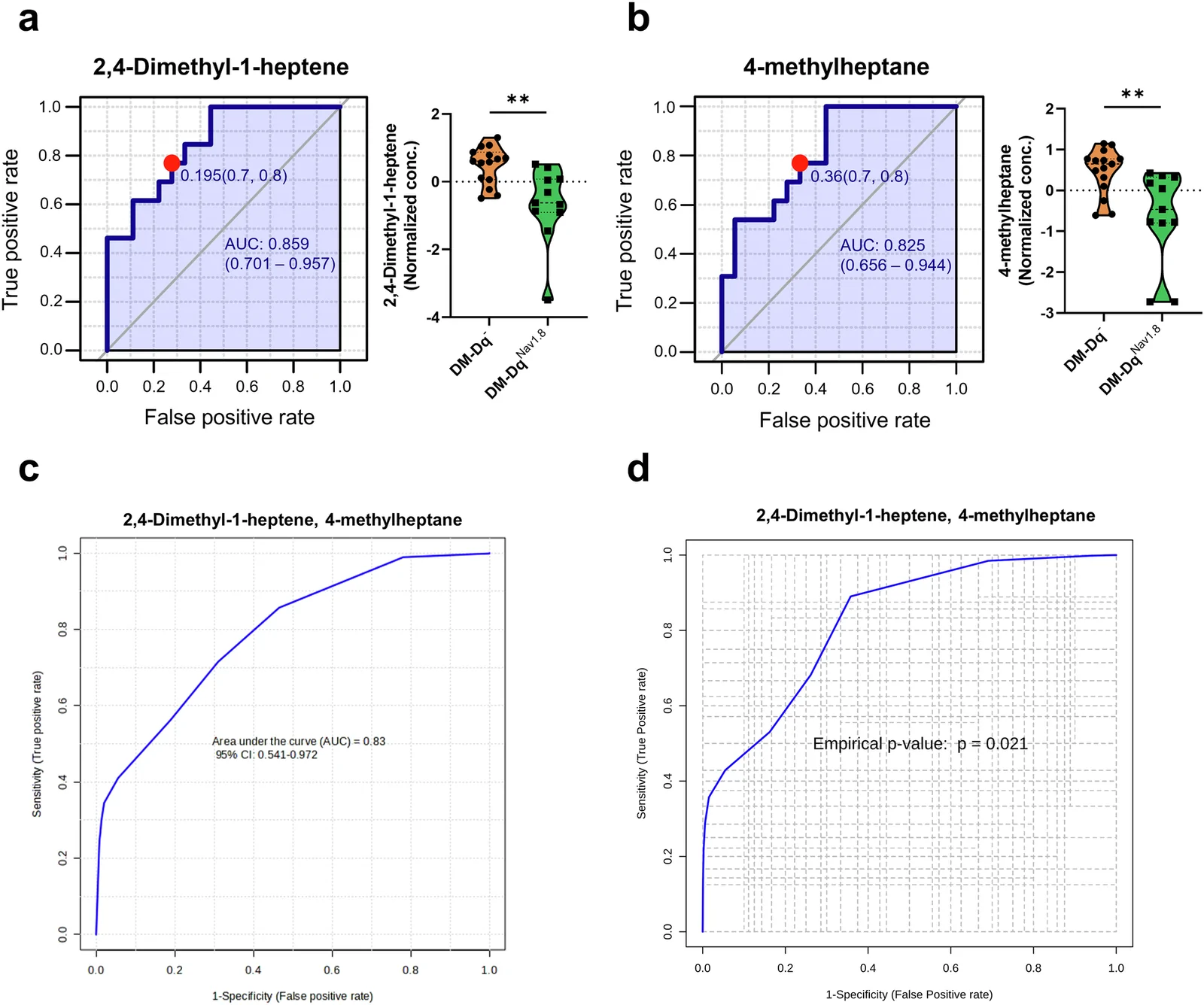
Development of VOC-based models to alleviate DG through Na_v_1.8-expressing neuronal activation. Receiver operating characteristic (ROC) curve testing of univariate models with 2,4-dimethyl-1-heptene (part **a**) or 4-methylheptane (part **b**) and their normalized concentrations in the DM-Dq^−^ and DM-Dq^Nav1.8^ mice. **c**, ROC curve of a multivariate model using a Random Forest algorithm with 2,4-dimethyl-1-heptene and 4-methylheptane. **d**, Validation of the multivariate model based on 2,4-dimethyl-1-heptene and 4-methylheptane using 1000 permutation tests (empirical *P* = 0.021). AUC, area under the curve; CI, confidence interval; DM, diabetes mellitus; DG, diabetic gastroparesis.

To further evaluate whether the breath VOCs identified from the comparison between DM-Dq^Nav1.8^ and DM-Dq^−^ mice could serve as treatment-response biomarkers of DG, we built classification models based on VOCs that distinguished between these two groups and assessed their discriminatory performance. VOCs with a VIP score > 1.0 from the breath analysis of the DM-Dq^Nav1.8^ and DM-Dq^−^ mice were identified as potential classification model features, excluding VOCs known to be exogenous. On the basis of these results, 2,4-dimethyl-1-heptene and 4-methylheptane were selected for both univariate and multivariate ROC curve analyses. In both cases, the concentrations of each VOC were higher in DM-Dq^−^ than in DM-Dq^Nav1.8^ mice (Fig. 5a,b). Univariate ROC curve analyses revealed that these VOCs performed well as binary classifiers in discriminating between DM-Dq^Nav1.8^ and DM-Dq^−^ mice, with AUC values of 0.859 for 2,4-dimethyl-1-heptene and 0.825 for 4-methylheptane. Multivariate ROC analysis, which used both VOCs as predictors, showed that the combination of these two VOCs effectively served as a classification model (Fig. 5c). The classification model for DG with 2,4-dimethyl-1-heptene and 4-methylheptane was further validated by a permutation test with 1000 iterations (Fig. 5d). Because the different patterns of breath VOCs observed in DM-Dq^Nav1.8^ and DM-Dq^−^ mice likely reflect physiological changes associated with improved gastric motility or other effects due to the stimulation of Na_v_1.8-expressing neurons, these classification models could potentially serve as tools for tracking treatment outcomes for DG and the alleviation of delayed gastric emptying symptoms.

## Discussion

In this study, we provide proof-of-concept evidence for a potential therapeutic strategy to ameliorate diabetes-induced gastric motility impairment and identify candidate biomarkers for monitoring this condition. Gastroparesis-like phenotypes were reproduced in mice with diabetes lasting more than 8 weeks, and our findings indicate that delayed gastric emptying can be counteracted in these animals by stimulating Na_v_1.8-expressing neurons. Exhaled breath VOC profiles differed by disease status and intervention: in particular, some VOCs, such as 1-butanol, 4-methylheptane, and 2,4-dimethyl-1-heptene, changed markedly when gastric motility improved following Na_v_1.8-expressing neuronal activation. Finally, using selected breath VOCs as exploratory biomarkers in this mouse model, we constructed preliminary classification models for monitoring DG.

The DG mouse model used in this study exhibited characteristic molecular alterations, including reduced expression of the ICC marker c-Kit under diabetic conditions. This reduction reflects impaired ICC function, which is crucial for maintaining gastrointestinal motility through pacemaker activity^27–30^. HO-1, an indicator of oxidative stress with cytoprotective properties^31,32^, was not upregulated in our DG model. This observation is consistent with previous studies in non-obese diabetic rodent models, in which the absence of HO-1 induction was associated with delayed gastric emptying and increased oxidative stress^23^. To validate our DG model and assess the therapeutic effects of Na_v_1.8-expressing neuronal stimulation, we used complementary methodologies, including the AAP absorption test as an indirect measure of gastric motility and IVIS for non-invasive, real-time imaging. Together, these approaches provide robust experimental support for the therapeutic potential of modulating Nav1.8-expressing neurons in gastroparesis.

Previous clinical studies have demonstrated that mutations in the *SCN10A* gene, which encodes Na_v_1.8, are associated with peripheral neuropathy and severe gastroparesis^16,33^. Additionally, genetic polymorphisms of *SCN10A* are closely linked to gastrointestinal dysfunction, particularly functional dyspepsia^17^. Recent studies have further shown that Na_v_1.8-expressing neurons are involved in the function of the enteric nervous system, suggesting a role for these neurons in gastrointestinal digestion and sensation^34,35^. Although these associations have been reported, direct regulation of gastric motility by Na_v_1.8-expressing neuronal activity has not been previously demonstrated. In this study, we used chemogenetic activation of Na_v_1.8-expressing neurons. Although this approach does not directly alter Na_v_1.8 function, it markedly ameliorated the gastroparesis phenotype. This observation that stimulation of Na_v_1.8-expressing neurons accelerated gastric motility under both DM and ND conditions suggests that these neurons represent a promising therapeutic target for gastroparesis.

Breath VOCs have been extensively investigated across a range of human disorders owing to their non-invasive nature and potential utility for early disease detection^8^. Some studies have suggested that breath VOCs may serve as potential biomarkers for various pathological conditions, such as cancers, infections, and liver diseases^36,37^. In our current study, we analyzed breath VOC patterns in mouse models of DM and DG. Moreover, we identified specific VOCs that differed significantly following modulation of Na_v_1.8-expressing neurons. These changes were associated with improved gastric emptying, suggesting that these compounds could serve as potential biomarkers for monitoring DG treatment.

The 1-butanol levels in the breath VOCs were significantly higher in the DM-WT mice than in the ND-WT mice (Fig. 2d). However, after stimulation of Na_v_1.8-expressing neurons, the 1-butanol levels were significantly lower in the DM-Dq^Nav1.8^ mice than in the DM-Dq^−^ mice (Fig. 4c,d). Previous studies have reported that experimentally induced systemic inflammation in male Sprague-Dawley rats increases 1-butanol levels in exhaled breath^38^. Given that increased inflammation is associated with diabetes, the elevated 1-butanol levels observed in our analyses may reflect this association. Additionally, in our current experiments, the 1-butanol levels in ND-WT mice decreased from 3 to 12 weeks (Supplementary Figs. 5c,d and 6), consistent with findings from a previous human study in which breath 1-butanol levels are negatively correlated with age^39^. Taken together, past evidence and our present results suggest that 1-butanol is a VOC that varies with both diabetes duration and aging. Furthermore, the decreased 1-butanol levels observed in our DG mice with improved gastric emptying support its potential utility as a biomarker for gastrointestinal motor function.

In addition to 1-butanol, several other VOCs were identified in our study as discriminative markers between DM-Dq^−^ and DM-Dq^Nav1.8^ mice, including styrene, 2,4-dimethyl-1-heptene, 4-methylheptane, and 2,4-dimethyl-1-decene. These VOCs also showed significant differences following neuronal stimulation within the ND group, suggesting a strong association with gastric motility. To evaluate their potential as biomarkers for the gastroparesis phenotype and for monitoring DG treatment, we developed multiple classification models. We assessed their performance in our DG mouse model using ROC analyses. During feature selection, exogenous compounds such as styrene were excluded from the classification models. Although 1-butanol could significantly discriminate between DM-Dq^−^ and DM-Dq^Nav1.8^ mice (Supplementary Fig. 10a), it was not included in the final model owing to its relatively low univariate ROC AUC value (Supplementary Fig. 11a) and its failure to improve model reliability when combined with other strong features (Supplementary Fig. 11b). 2-Ethylhexan-1-ol, which was elevated in diabetic DG mice (Fig. 2d), showed only a modest difference between DM-Dq^−^ and DM-Dq^Nav1.8^ mice following activation of Na_v_1.8-expressing neurons (FC = 1.19). Furthermore, ROC analysis demonstrated relatively limited discriminative performance compared with other candidate VOCs (Supplementary Fig. 11c,d); therefore, 2-ethylhexan-1-ol was excluded from the final classification model. Through this systematic analysis, we identified an optimal classification model comprising two VOCs — 2,4-dimethyl-1-heptene and 4-methylheptane — for assessing effects on gastric emptying. These findings support the potential utility of breath VOC-based models for monitoring DG progression and treatment response.

There are several notable limitations to our study. DG occurs in both type 1 diabetes (T1DM) and type 2 diabetes (T2DM); our study employed an STZ-induced diabetes model to mimic T1DM complications, given that the prevalence of gastroparesis in individuals with T1DM is more than fivefold higher than in T2DM^40^. The pathophysiological mechanisms underlying DG in T2DM may differ. Therefore, future investigations should include T2DM models to provide a more comprehensive understanding of DG and to develop effective treatments. Na_v_1.8 is expressed in both vagal and spinal afferents, complicating precise manipulation of Na_v_1.8-expressing neurons to enhance gastric motility in humans. Further studies are thus required to identify which afferent pathways are critical for regulating gastric motility. The activation of Na_v_1.8-expressing neurons may also have effects beyond improving gastric emptying, making it difficult to delineate the relationship between gastric motility and the filtered VOCs. As DM can lead to various complications in humans, it is important to be cautious when attributing each VOC change to a single phenomenon. Another limitation of our present analysis is the paucity of comprehensive reference data for breath VOCs in mice and other rodents and the currently limited understanding of the correlation between exhaled VOCs in humans and those in mice. Additionally, our study was conducted exclusively in male mice, which limits the generalizability of our findings to females. Future studies should include both sexes to account for potential sex-based differences in gastric motility and exhaled VOC profiles. In this study, the term “treatment” refers to short-term symptomatic relief of delayed gastric emptying, not to modification of the underlying disease. Accordingly, further studies are needed to evaluate long-term effects and potential disease-modifying outcomes.

Despite these limitations, our study demonstrates the clinical potential of breath VOCs for assessing diabetes and its complications. This area has been little studied to date in animal models. Furthermore, no research has yet investigated breath VOCs in relation to gastric motility and to their improvement through modulation of Na_v_1.8-expressing neurons. Notably, the breath VOCs detected by our current analyses are also known constituents of human breath, suggesting that corresponding VOCs can be detected in human biological samples.

For future clinical applications, it will be crucial to establish robust quantification methods using standardized reference compounds and to define normal and pathological ranges for accurate assessment of DG status. To improve the reliability and applicability of our findings, additional breath samples must be collected and repeatedly analyzed to develop a robust detection model. In addition, because our current study was conducted using mouse models, a comprehensive human breath database for diabetes must be established and analyzed before clinical application. Although the specific VOCs serving as potential biomarkers for DG may vary across experimental settings, this proof-of-concept study demonstrates the methodological feasibility and translational potential of breath VOC-based DG assessment. Our results imply that Na_v_1.8-expressing neuron-driven enhancement of afferent pathways can promote gastric emptying even in the absence of diabetes. Given that prokinetic therapies are widely used in patients with non-diabetes with functional dyspepsia and other conditions associated with delayed gastric emptying, our findings suggest that selectively targeting Na_v_1.8-expressing neurons may represent a therapeutic strategy with potential broader applicability, while minimizing adverse off-target effects. Although the specific cells targeted by Na_v_1.8 in humans remain unclear, our current results indicate that Na_v_1.8-expressing neurons contribute to improved gastric motility. This suggests the potential to develop medical devices that use electrical stimulation of Na_v_1.8-expressing neurons to enhance gastric motility in patients with gastroparesis.

## Supplementary information

Supplementary information

## Data Availability

The metabolomic dataset generated in this study has been deposited in MetaboLights under accession number MTBLS12847. Other data are available from the lead contact upon reasonable request.

## References

[1] Azpiroz, F. & Malagelada, C. Diabetic neuropathy in the gut: pathogenesis and diagnosis. *Diabetologia* **59**, 404–408 (2016). 10.1007/s00125-015-3831-1 26643877

[2] Kuznik, E., Dudkowiak, R., Adamiec, R. & Poniewierka, E. Diabetic autonomic neuropathy of the gastrointestinal tract. *Prz. Gastroenterol* **15**, 89–93 (2020). 10.5114/pg.2020.95554 PMC7294968 32550939

[3] Parkman, H. P., Hasler, W. L. & Fisher, R. S. American Gastroenterological, American Gastroenterological Association technical review on the diagnosis and treatment of gastroparesis. *Gastroenterology* **127**, 1592–1622 (2004). 10.1053/j.gastro.2004.09.055 15521026

[4] Ghazanfar, H. et al. Diagnostic modalities used in diagnosing gastroparesis: a clinical review. *Cureus* **14**, e30540 (2022). 10.7759/cureus.30540 PMC9675943 36415382

[5] Lacy, B. E., Tack, J. & Gyawali, C. P. AGA clinical practice update on management of medically refractory gastroparesis: expert review. *Clin Gastroenterol Hepatol* **20**, 491–500 (2022). 10.1016/j.cgh.2021.10.038 34757197

[6] Vohra, I., Gopakumar, H. & Adler, D. G. G-POEM for gastroparesis: past, present and future. *Curr. Opin. Gastroenterol* **39**, 390–396 (2023). 10.1097/MOG.0000000000000950 37265234

[7] Bharucha, A. E., Kudva, Y. C. & Prichard, D. O. Diabetic gastroparesis. *Endocr. Rev.* **40**, 1318–1352 (2019). 10.1210/er.2018-00161 PMC6736218 31081877

[8] Moura, P. C., Raposo, M. & Vassilenko, V. Breath volatile organic compounds (VOCs) as biomarkers for the diagnosis of pathological conditions: a review. *Biomed. J* **46**, 100623 (2023). 10.1016/j.bj.2023.100623 PMC10339195 37336362

[9] Smolinska, A. et al. Volatile metabolites in breath strongly correlate with gut microbiome in CD patients. *Anal. Chim. Acta* **1025**, 1–11 (2018). 10.1016/j.aca.2018.03.046 29801597

[10] Parkman, H. P. Assessment of gastric emptying and small-bowel motility: scintigraphy, breath tests, manometry, and SmartPill. *Gastrointest Endosc. Clin. N Am.* **19**, 49–55 (2009). vi. 10.1016/j.giec.2008.12.003 19232280

[11] Sangnes, D. A., Softeland, E., Teigland, T. & Dimcevski, G. Comparing radiopaque markers and ^13^C-labelled breath test in diabetic gastroparesis diagnostics. Clin. Exp. Gastroenterol 12, 193–201 (2019). 10.2147/CEG.S200875 PMC6511612 31190946

[12] Stirling, L. C. et al. Nociceptor-specific gene deletion using heterozygous NaV1.8-Cre recombinase mice. *Pain* **113**, 27–36 (2005). 10.1016/j.pain.2004.08.015 15621361

[13] Ye, P. et al. Functional up-regulation of Nav1.8 sodium channel on dorsal root ganglia neurons contributes to the induction of scorpion sting pain. *Acta Biochim. Biophys. Sin (Shanghai)* **48**, 132–144 (2016). 10.1093/abbs/gmv123 26764239

[14] Akopian, A. N., Sivilotti, L. & Wood, J. N. A tetrodotoxin-resistant voltage-gated sodium channel expressed by sensory neurons. *Nature* **379**, 257–262 (1996). 10.1038/379257a0 8538791

[15] Gautron, L. et al. Genetic tracing of Nav1.8-expressing vagal afferents in the mouse. *J Comp. Neurol* **519**, 3085–3101 (2011). 10.1002/cne.22667 PMC3306808 21618224

[16] Dabby, R. et al. Painful small fiber neuropathy with gastroparesis: a new phenotype with a novel mutation in the SCN10A gene. *J Clin. Neurosci* **26**, 84–88 (2016). 10.1016/j.jocn.2015.05.071 26711856

[17] Arisawa, T. et al. Genetic polymorphisms of SCN10A are associated with functional dyspepsia in Japanese subjects. *J Gastroenterol* **48**, 73–80 (2013). 10.1007/s00535-012-0602-3 22618805

[18] Maida, A., Lovshin, J. A., Baggio, L. L. & Drucker, D. J. The glucagon-like peptide-1 receptor agonist oxyntomodulin enhances beta-cell function but does not inhibit gastric emptying in mice. *Endocrinology* **149**, 5670–5678 (2008). 10.1210/en.2008-0336 18669601

[19] Dargue, R. et al. The analysis of acetaminophen (paracetamol) and seven metabolites in rat, pig and human plasma by U(H)PLC-MS. *Bioanalysis* **12**, 485–500 (2020). 10.4155/bio-2020-0015 32343149

[20] Lee, H. S., Kim, N. Y. & Kim, J. A fatal case of mexiletine intoxication in a 25-year-old Korean man. *Foren. Toxicol* **38**, 517–522 (2020).

[21] Pang, Z. et al. MetaboAnalyst 5.0: narrowing the gap between raw spectra and functional insights. *Nucleic Acids Res.* **49**, W388–W396 (2021). 10.1093/nar/gkab382 PMC8265181 34019663

[22] Grover, M. et al. Clinical-histological associations in gastroparesis: results from the Gastroparesis Clinical Research Consortium. *Neurogastroenterol Motil.* **24**, 531–539.e249 (2012). 10.1111/j.1365-2982.2012.01894.x PMC3353102 22339929

[23] Choi, K. M. et al. Heme oxygenase-1 protects interstitial cells of Cajal from oxidative stress and reverses diabetic gastroparesis. *Gastroenterology* **135**, 2055–2064 (2008). 10.1053/j.gastro.2008.09.003 PMC2796242 18926825

[24] Browning, K. N. & Travagli, R. A. Central nervous system control of gastrointestinal motility and secretion and modulation of gastrointestinal functions. *Compr. Physiol.* **4**, 1339–1368 (2014). 10.1002/cphy.c130055 PMC4858318 25428846

[25] Gong, Y. et al. Effects of ghrelin on gastric distension sensitive neurons and gastric motility in the lateral septum and arcuate nucleus regulation. *J Gastroenterol* **49**, 219–230 (2014). 10.1007/s00535-013-0789-y 23525979

[26] Gong, Y. et al. Involvements of the lateral hypothalamic area in gastric motility and its regulation by the lateral septum. *Gen. Comp. Endocrinol* **194**, 275–285 (2013). 10.1016/j.ygcen.2013.09.022 24100167

[27] Huizinga, J. D., Zarate, N. & Farrugia, G. Physiology, injury, and recovery of interstitial cells of Cajal: basic and clinical science. *Gastroenterology* **137**, 1548–1556 (2009). 10.1053/j.gastro.2009.09.023 PMC2943431 19778538

[28] Habtezion, A. & Wiley, J. W. Diabetic gastroparesis: an emerging role for macrophages and heme oxygenase-1. *Gastroenterology* **138**, 2219–2223 (2010). 10.1053/j.gastro.2010.04.028 20434508

[29] Ordog, T., Takayama, I., Cheung, W. K., Ward, S. M. & Sanders, K. M. Remodeling of networks of interstitial cells of Cajal in a murine model of diabetic gastroparesis. *Diabetes* **49**, 1731–1739 (2000). 10.2337/diabetes.49.10.1731 11016458

[30] Sanders, K. M., Ordog, T., Koh, S. D., Torihashi, S. & Ward, S. M. Development and plasticity of interstitial cells of Cajal. *Neurogastroenterol Motil* **11**, 311–338 (1999). 10.1046/j.1365-2982.1999.00164.x 10520164

[31] Ryter, S. W., Alam, J. & Choi, A. M. Heme oxygenase-1/carbon monoxide: from basic science to therapeutic applications. *Physiol. Rev.* **86**, 583–650 (2006). 10.1152/physrev.00011.2005 16601269

[32] Abraham, N. G., Asija, A., Drummond, G. & Peterson, S. Heme oxygenase -1 gene therapy: recent advances and therapeutic applications. *Curr. Gene. Ther.* **7**, 89–108 (2007). 10.2174/156652307780363134 17430129

[33] Faber, C. G. et al. Gain-of-function Nav1.8 mutations in painful neuropathy. *Proc. Natl. Acad Sci. USA.* **109**, 19444–19449 (2012). 10.1073/pnas.1216080109 PMC3511073 23115331

[34] Bullich-Vilarrubias, C. et al. Nav1.8-expressing neurons control daily oscillations of food intake, body weight and gut microbiota in mice. *Commun Biol.* **7**, 219 (2024). 10.1038/s42003-024-05905-3 PMC10883928 38388698

[35] Romani-Perez, M., Bullich-Vilarrubias, C., Lopez-Almela, I. & Sanz, Y. The ablation of sensory neurons expressing the Nav1.8 sodium channel improves glucose homeostasis and amplifies the GLP-1 signaling in obese female mice. *Mol Nutr. Food Res.* **68**, e2300474 (2024). 10.1002/mnfr.202300474 38038153

[36] Zhou, M. et al. Exhaled breath and urinary volatile organic compounds (VOCs) for cancer diagnoses, and microbial-related VOC metabolic pathway analysis: a systematic review and meta-analysis. *Int. J Surg.* **110**, 1755–1769 (2024). 10.1097/JS9.0000000000000999 PMC10942174 38484261

[37] Moura, P. C., Raposo, M. & Vassilenko, V. Breath biomarkers in non-carcinogenic diseases. Clin. Chim. Acta. **552**, 117692 (2024). 10.1016/j.cca.2023.117692 38065379

[38] Albrecht, F. W. et al. Exhaled volatile organic compounds during inflammation induced by TNF-alpha in ventilated rats. *Metabolites* **10**, 245 (2020). 10.3390/metabo10060245 PMC7345252 32549262

[39] Koureas, M. et al. Target analysis of volatile organic compounds in exhaled breath for lung cancer discrimination from other pulmonary diseases and healthy persons. *Metabolites* **10**, 317 (2020). 10.3390/metabo10080317 PMC7464039 32756521

[40] Choung, R. S. et al. Risk of gastroparesis in subjects with type 1 and 2 diabetes in the general population. *Am J Gastroenterol* **107**, 82–88 (2012). 10.1038/ajg.2011.310 PMC3280088 22085818

