# Supplementary material for: Breath volatiles reflect diabetic gastroparesis and its recovery by activating Nav1.8-expressing neurons in mice
Source: Exp Mol Med. 2026 Sep 24;58(9):3214–25. doi: 10.1038/s12276-026-01824-x (PMC13633264; doi:10.1038/s12276-026-01824-x)
Supplement: Supplementary file 1 [file 12276_2026_1824_MOESM1_ESM.pdf]

Supplementary information

Breath volatiles reflect diabetic gastroparesis and its recovery by activating $Na_v1.8$-expressing neurons in mice

Running title: VOCs track recovery from gastroparesis

Seiyoung Hwang†, Evonne Kim†, Soyun Lee†, Daum Lee, Minjoo Park, Ho-Sun Lee,
Hyunsoo Chung*, Obin Kwon*

This file includes:

- Supplementary Materials and Methods
- Supplementary Figures 1 to 11

Supplementary Materials and Methods

Immunohistochemistry

Mice were anesthetized with Zoletil + Rompun and transcardially perfused with PBS. The brains were then harvested and post-fixed overnight at 4°C in 4% paraformaldehyde, dehydrated in a PBS-based 30% sucrose solution, and then coronally sliced into 25 µm-thick sections using a cryostat (Epredia™ CryoStar NX50, Thermo Fisher Scientific). Brain slices were washed 5 times in PBS, blocked with 5% donkey serum in PBS containing 0.3% Triton X-100 for 1 hour at room temperature, and then incubated with rabbit monoclonal anti-c-Fos primary antibody (1:1000, #2250, Cell Signaling) overnight at 4°C. The following day, after 4 washes in PBS, slides were incubated with a secondary antibody [donkey anti-rabbit Alexa Fluor 488 (1:2000, A21206, Invitrogen, Waltham, MA, USA)] for 2 hours at room temperature. After several further washes, slides were incubated with DAPI (0.5 mg/ml, D9542, Sigma-Aldrich) for 5 minutes at room temperature and mounted using a fluorescent mounting medium (S3023, DAKO, Glostrup, Denmark). Images were acquired using a ZEISS LSM980 Airyscan2 confocal microscope, and cell counts were performed with the ImageJ software.

Supplementary figures

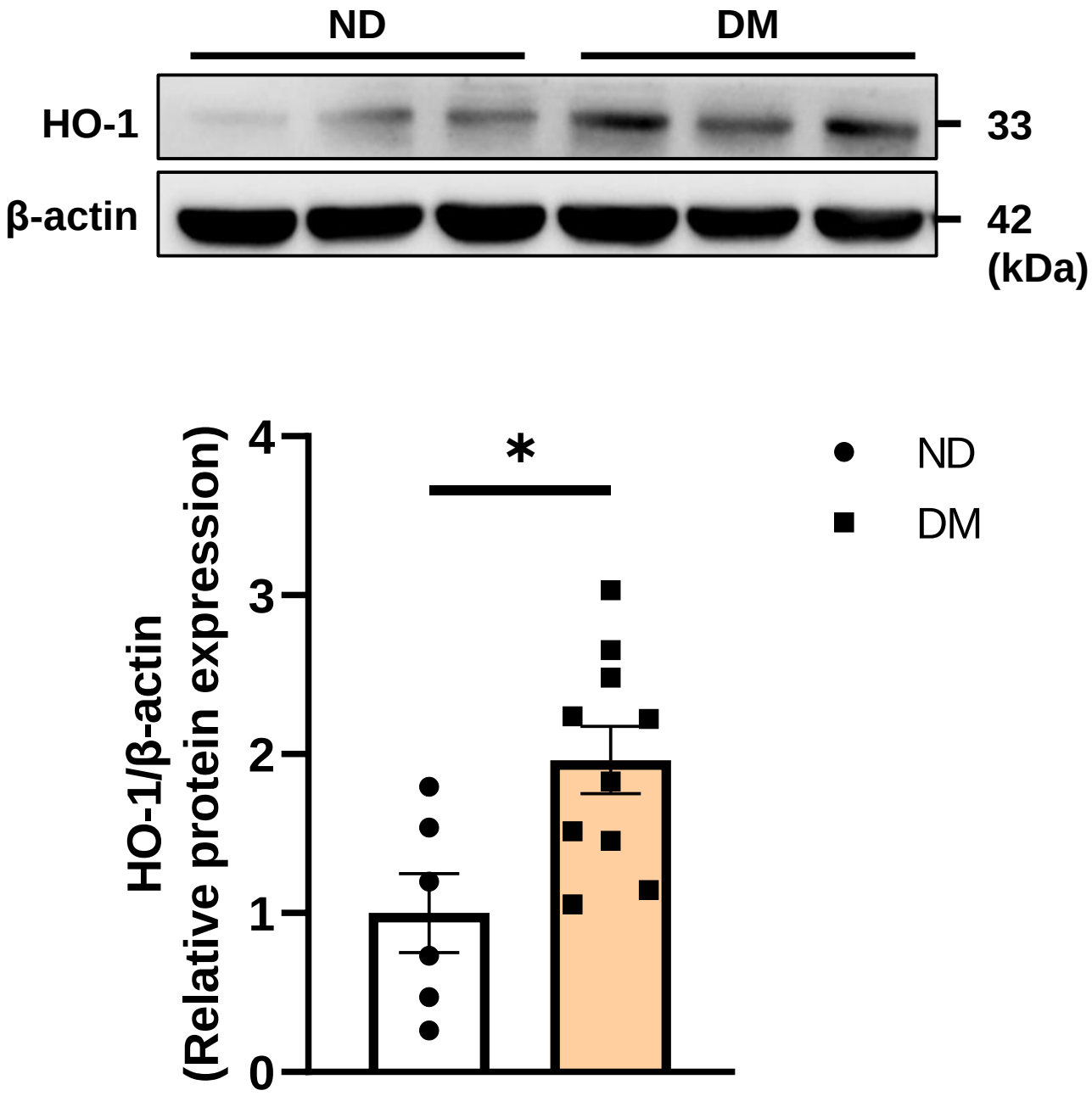

Supplementary Fig. 1. Increased HO-1 expression in the stomach at the early stage of diabetes. Representative immunoblots and quantification of relative HO-1 protein expression in stomach tissue at 4 weeks after diabetes induction. Data are presented as mean ± SEM (n=6 ND and n=10 DM). Statistical analysis was conducted using an unpaired t-test. * $P < .05$.

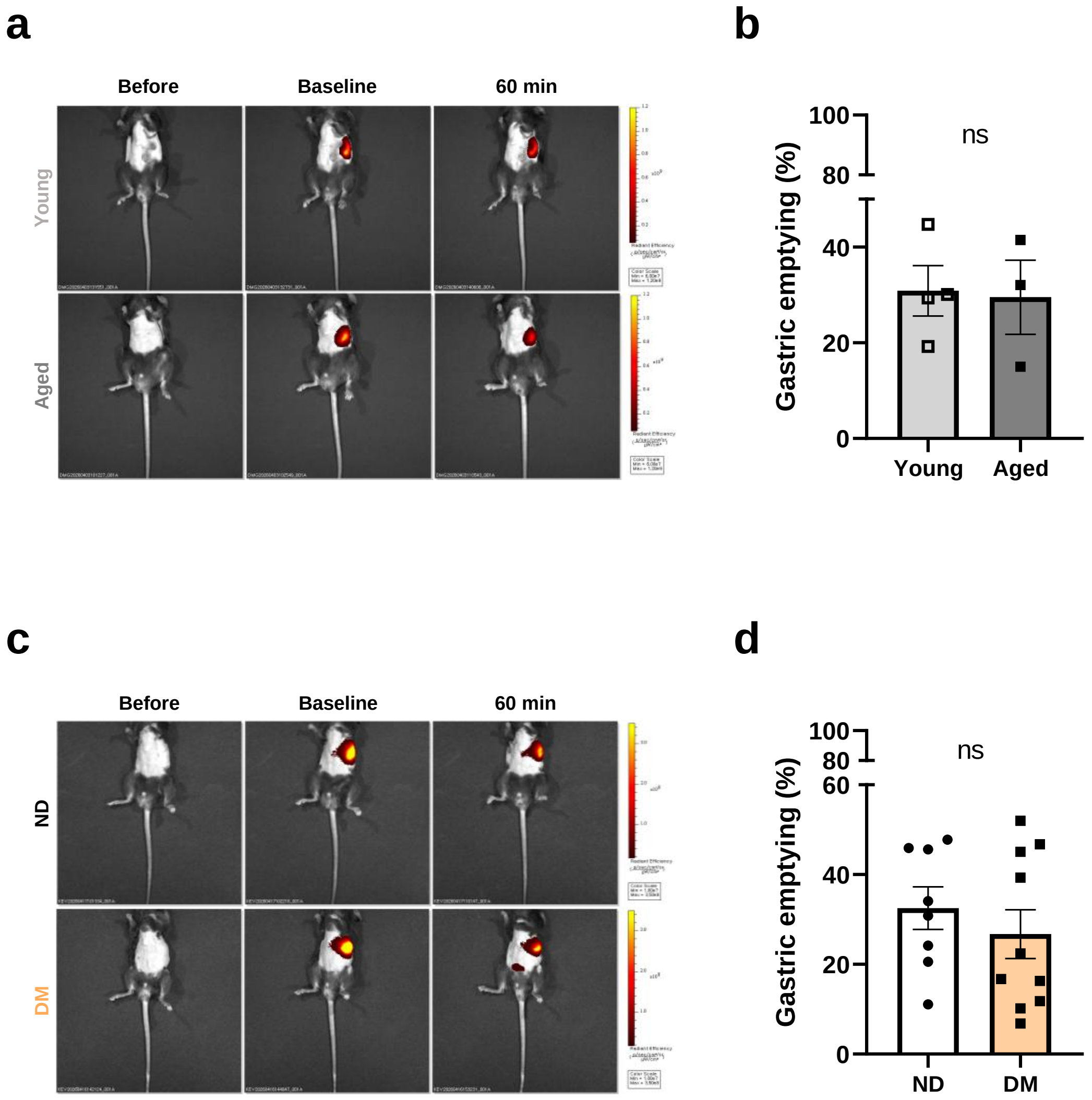

Supplementary Fig. 2. Gastric emptying across age-related maturation and diabetes progression. (a-b) Gastric emptying in young (10-week-old) and aged (40-week-old) male wild-type (WT) mice, assessed using the IVIS Spectrum. (a) Representative images and (b) gastric emptying ratio (%). (c-d) Gastric emptying in male WT non-diabetic (ND) and diabetic (DM; 3 weeks after DM induction) mice, assessed using the IVIS Spectrum. (c) Representative images and (d) gastric emptying ratio (%). Data are presented as mean ± SEM (n=4 Young, n=3 Aged, n=8 ND and n=10 DM). Statistical analysis was conducted using an unpaired t-test. min, minutes; ns, not significant.

a

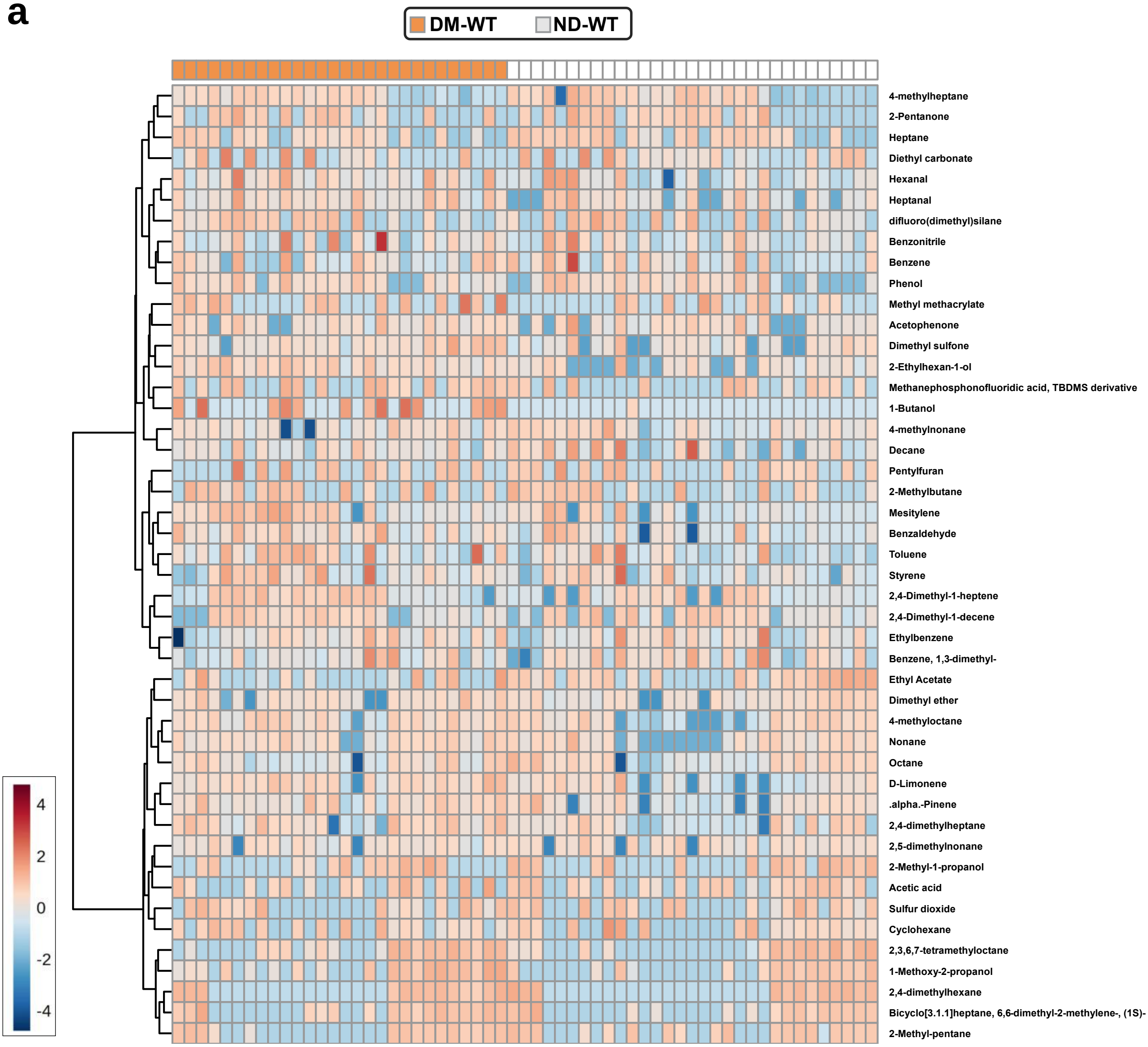

b

12 weeksDM - WT / ND - WT		
**Name of volatile organic compounds**	**Log_2(Fold Change)**	**p-value**
**1-Butanol**	4.6945	0.0000823
**2-Ethylhexan-1-ol**	1.039	0.000123

Supplementary Fig. 3. Contrasting breath VOC patterns in DM-WT and ND-WT mice at 12 weeks after diabetes induction. (a) Hierarchical clustering heatmap of breath VOCs generated using Ward's clustering method (gray, ND-WT; orange, DM-WT). (b) Table of breath VOCs identified as statistically meaningful ($|log_2FC| \geq 1.0$, $P < .05$) from a comparison of DM-WT and ND-WT mice.

a

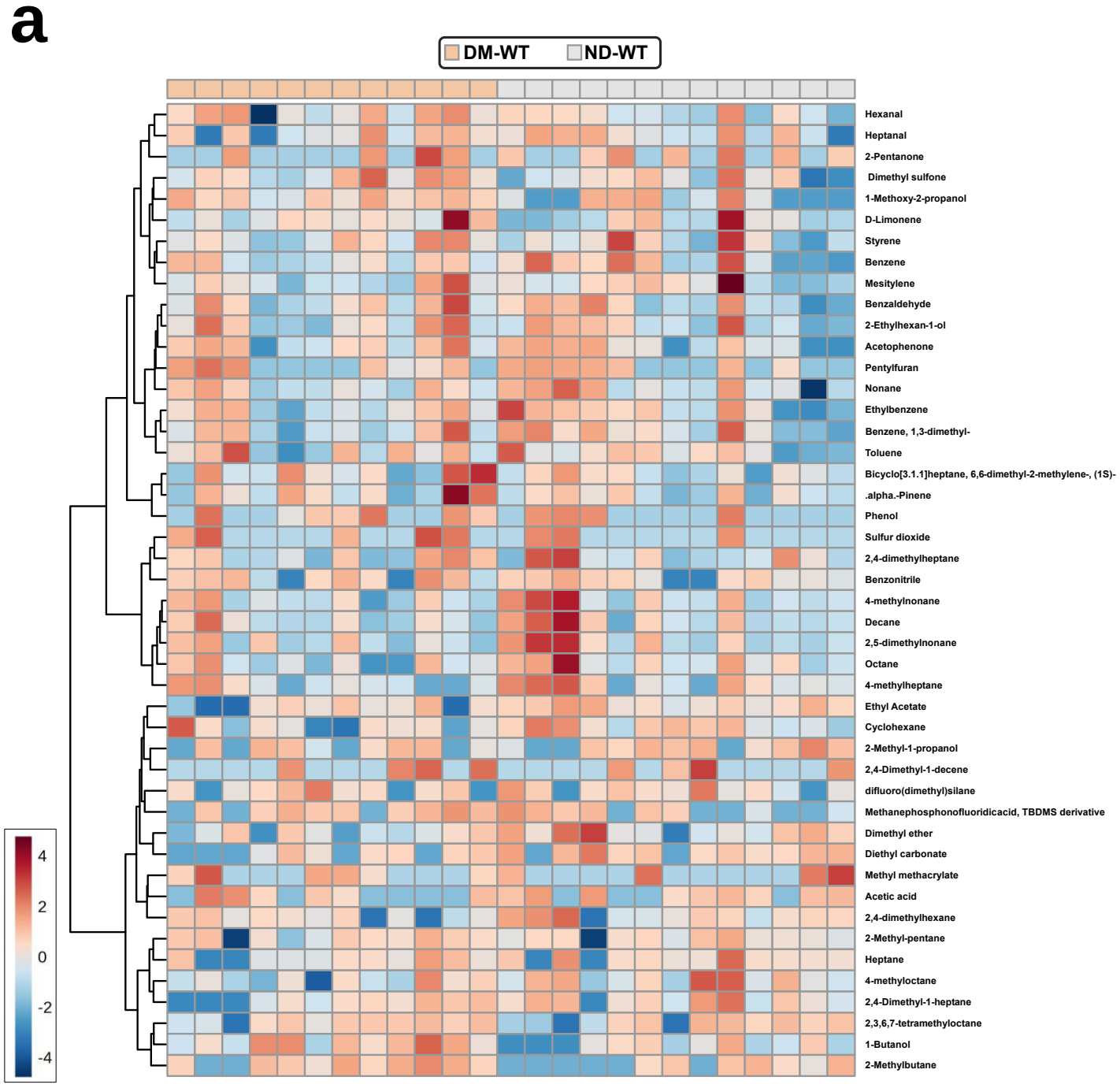

b

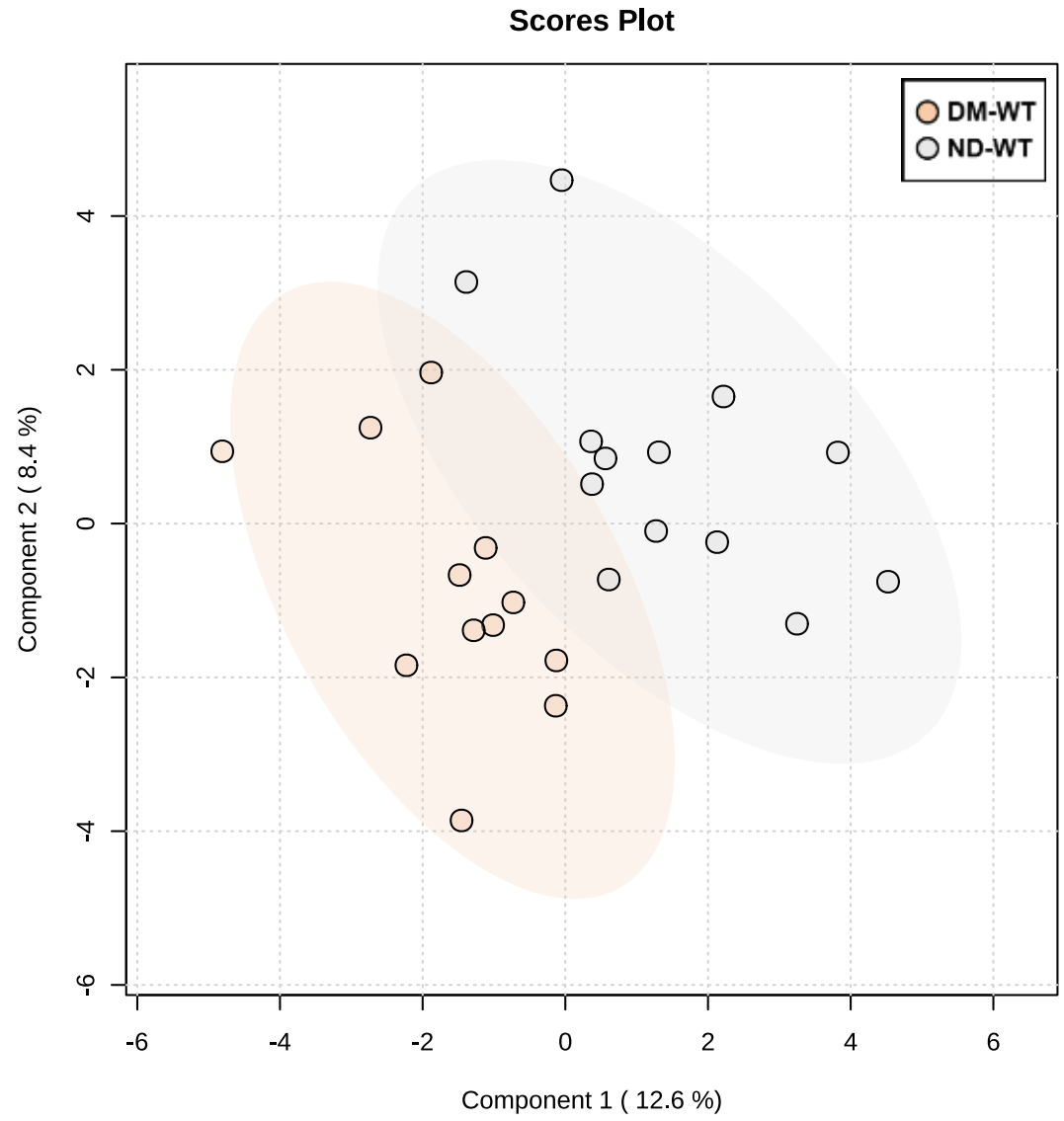

c

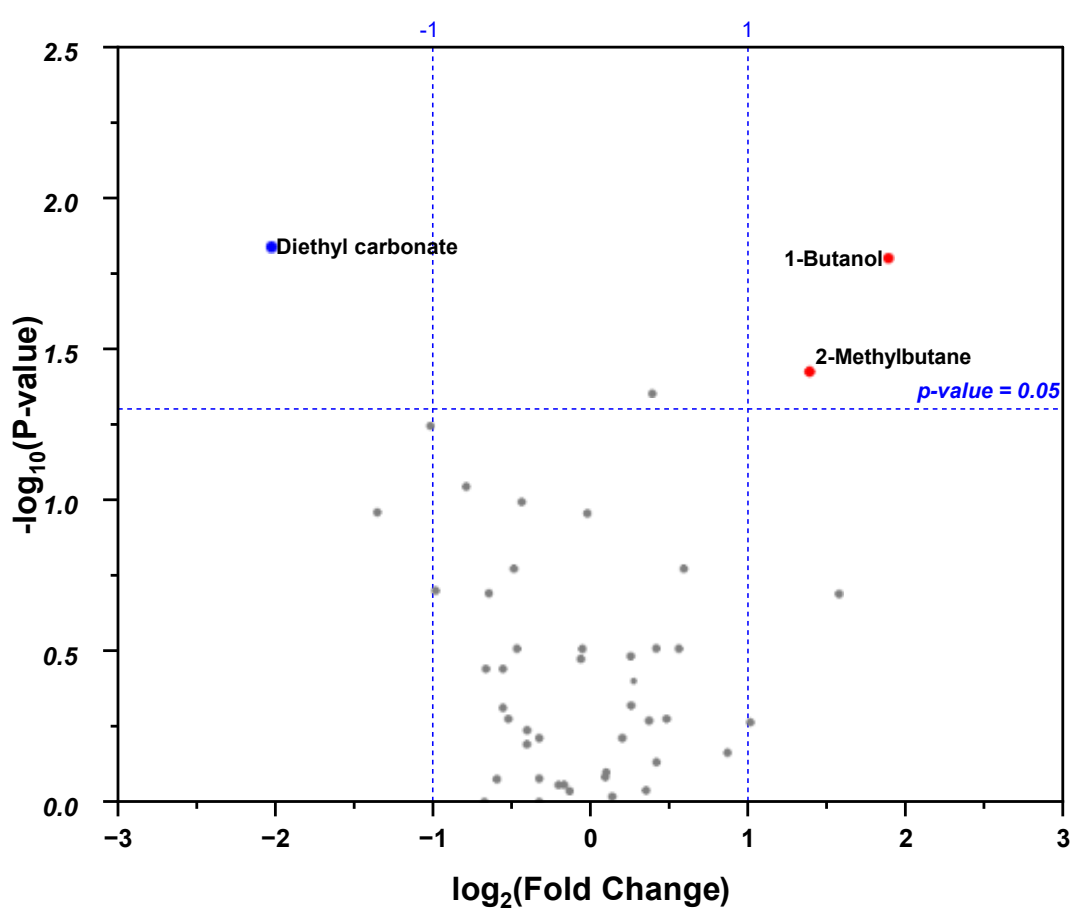

d

3 weeksDM - WT / ND - WT		
**Name of volatile organic compounds**	**Log_2(Fold Change)**	**p-value**
**Diethyl carbonate**	-2.0256	0.01446
**1-Butanol**	1.8866	0.015869
**2-Methylbutane**	1.3895	0.037399

Supplementary Fig. 4. Contrasting breath VOC patterns in the DM-WT and ND-WT mice at 3 weeks after diabetes induction. (a) Hierarchical clustering heatmap of breath VOCs generated using Ward's clustering method (light gray, ND-WT; light orange, DM-WT). (b) PLS-DA plot showing classification of VOC patterns in DM-WT and ND-WT mice. (c) Volcano plot showing differential breath VOCs between DM-WT and ND-WT mice identified using the Wilcoxon rank-sum test ($|log_2FC| \geq 1.0$, $P < .05$). (d) Table of statistically altered breath VOCs between DM-WT and ND-WT mice at 3 weeks after DM induction ($|log_2FC| \geq 1.0$, $P < .05$).

a

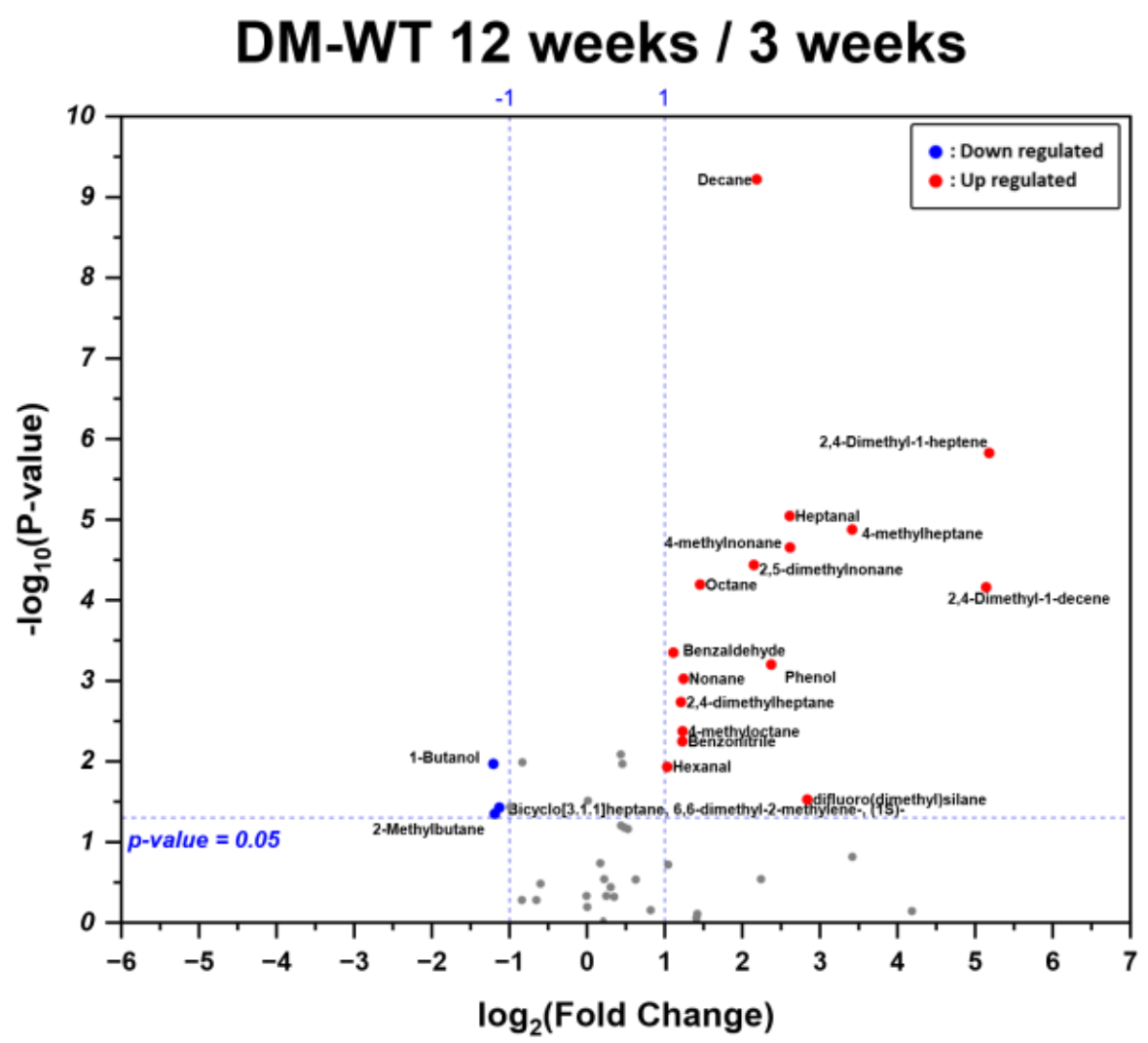

b

DM-WT 12 weeks / 3 weeks		
**Name of volatile organic compounds**	**Log_2(Fold Change)**	**p-value**
**Decane**	2.1901	0.0000000000000682
**4-methylheptane**	3.4168	0.00000002
**2,4-Dimethyl-1-heptene**	5.1831	0.0000000347
**Heptanal**	2.6153	0.00000202
**2,4-Dimethyl-1-decene**	5.1445	0.00000488
**2,5-dimethylnonane**	2.6193	0.0000431
**Benzaldehyde**	1.1174	0.00016
**4-methylnonane**	2.1529	0.000865
**Phenol**	2.3763	0.001058
**Benzonitrile**	1.2314	0.001141
**1-Butanol**	-1.2034	0.002256
**Octane**	1.4589	0.003232
**Hexanal**	1.0368	0.00407
**Bicyclo[3.1.1]heptane, 6,6-dimethyl-2-methylene-, (1S)-**	-1.1259	0.005482
**Nonane**	1.2454	0.01955
**2-Methylbutane**	-1.1871	0.037725
**2,4-dimethylheptane**	1.2147	0.038674
**4-methyloctane**	1.2347	0.067327
**difluoro(dimethyl)silane**	2.8409	0.076583

c

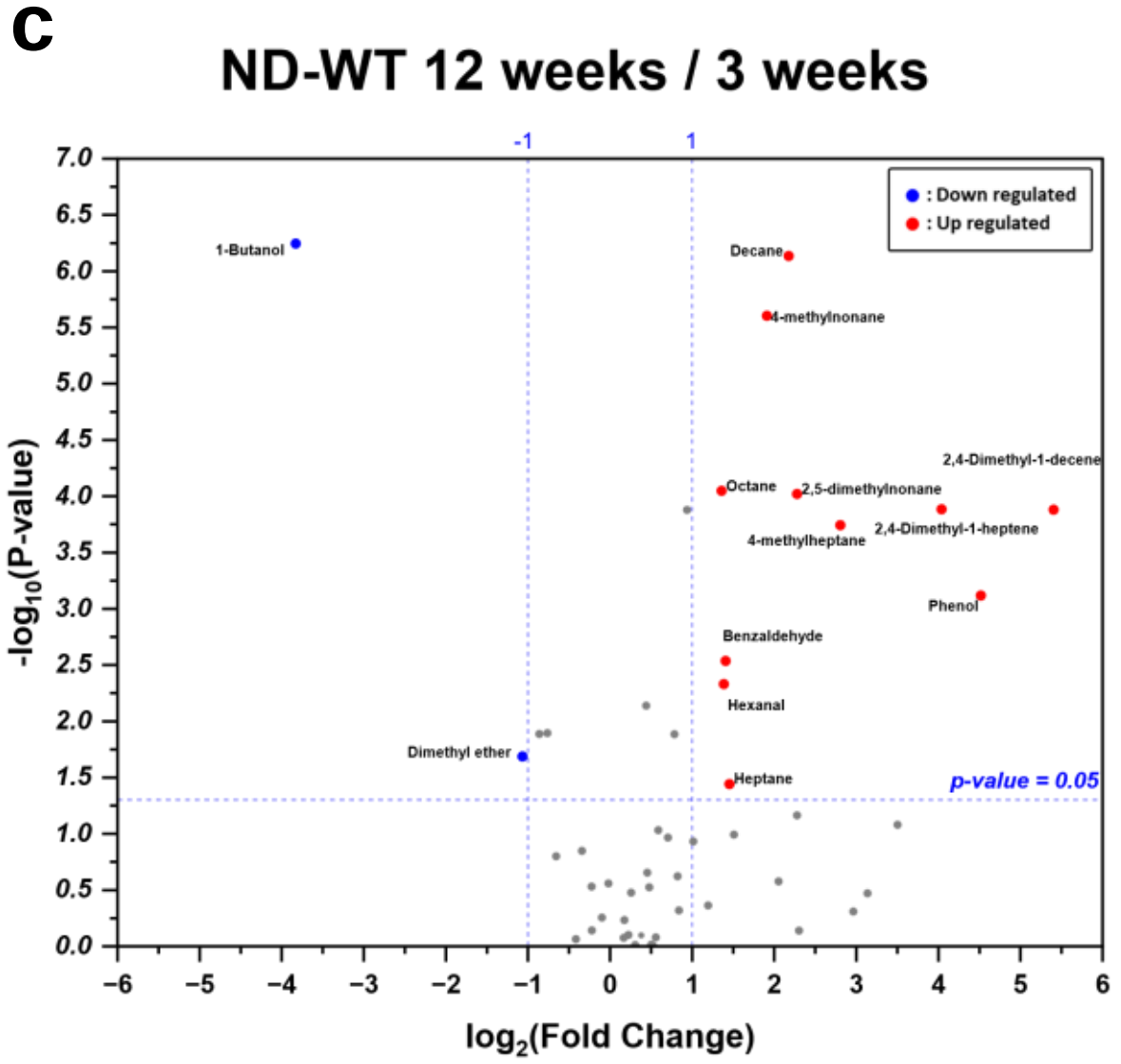

d

ND-WT 12 weeks / 3 weeks		
**Name of volatile organic compounds**	**Log_2(Fold Change)**	**p-value**
**1-Butanol**	-3.827	0.000000572
**Decane**	2.1798	0.000000737
**4-methylnonane**	1.9133	0.0000025
**Octane**	1.3614	0.0000899
**2,5-dimethylnonane**	2.2795	0.0000957
**2,4-Dimethyl-1-heptene**	4.0409	0.000131
**2,4-Dimethyl-1-decene**	5.4061	0.000132
**4-methylheptane**	2.8097	0.000182
**Phenol**	4.5218	0.000766
**Benzaldehyde**	1.4111	0.002924
**Hexanal**	1.3881	0.004682
**Dimethyl ether**	-1.0628	0.02067
**Heptane**	1.458	0.036319

Supplementary Fig. 5. Comparison of breath VOC profiles in DM-WT mice at 3 and 12 weeks after diabetes induction and in age-matched ND-WT mice. (a) Volcano plot showing differential breath VOCs in DM-WT mice identified using the Wilcoxon rank-sum tests and (b) table of statistically significant breath VOCs ($|log_2FC| \geq 1.0$, $P < .05$) in the DM-WT mice. (c) Volcano plot showing differential breath VOCs identified using the Wilcoxon rank-sum tests and (d) table of statistically significant breath VOCs ($|log_2FC| \geq 1.0$, $P < .05$) in the ND-WT mice.

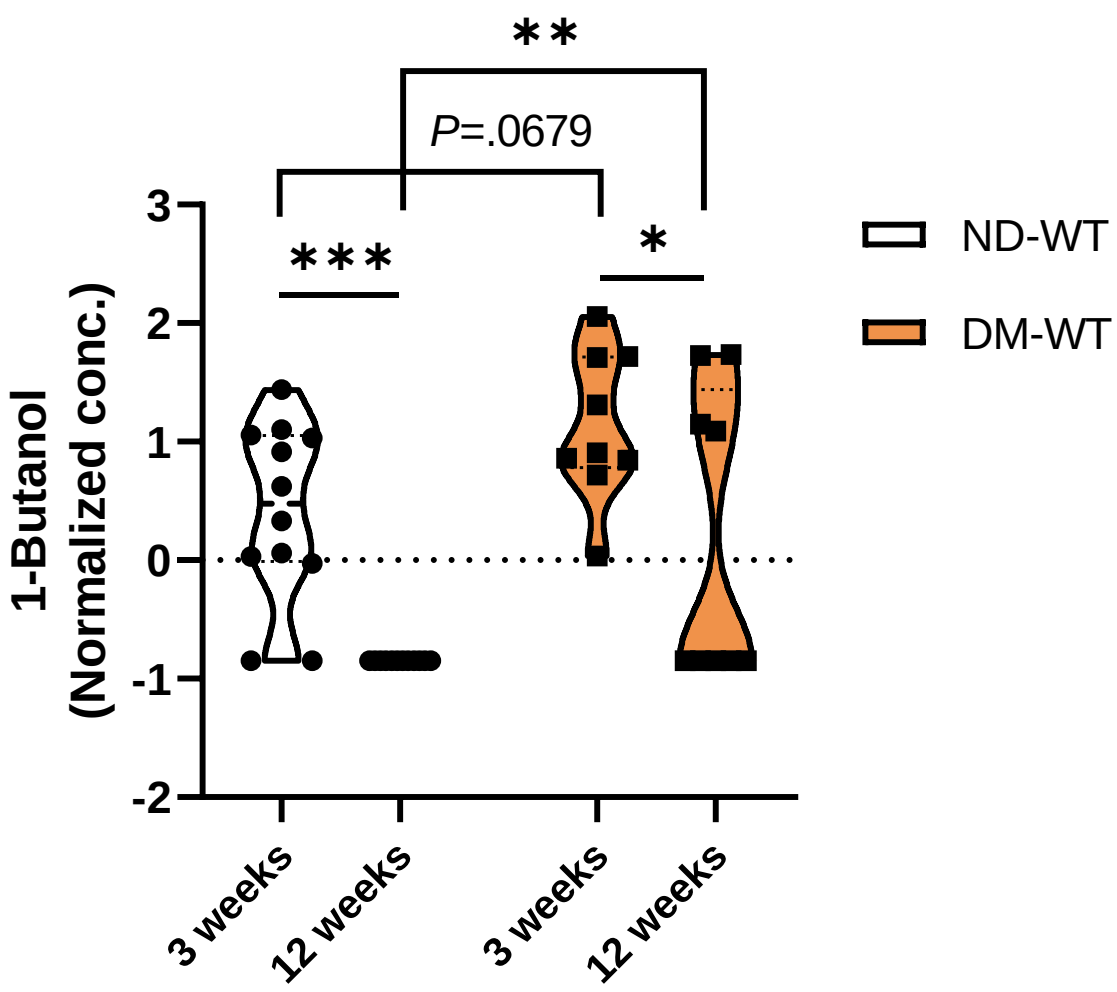

Supplementary Fig. 6. Normalized concentrations of 1-butanol for DM-WT mice at 3 and 12 weeks after diabetes induction and in age-matched ND-WT mice. Statistical analysis was performed using two-way repeated-measures ANOVA followed by Sidak's post-hoc test. Data are presented as mean ± SEM (n=12 ND-WT and n=9 DM-WT); * $P < .05$. ** $P < .01$. *** $P < .001$.

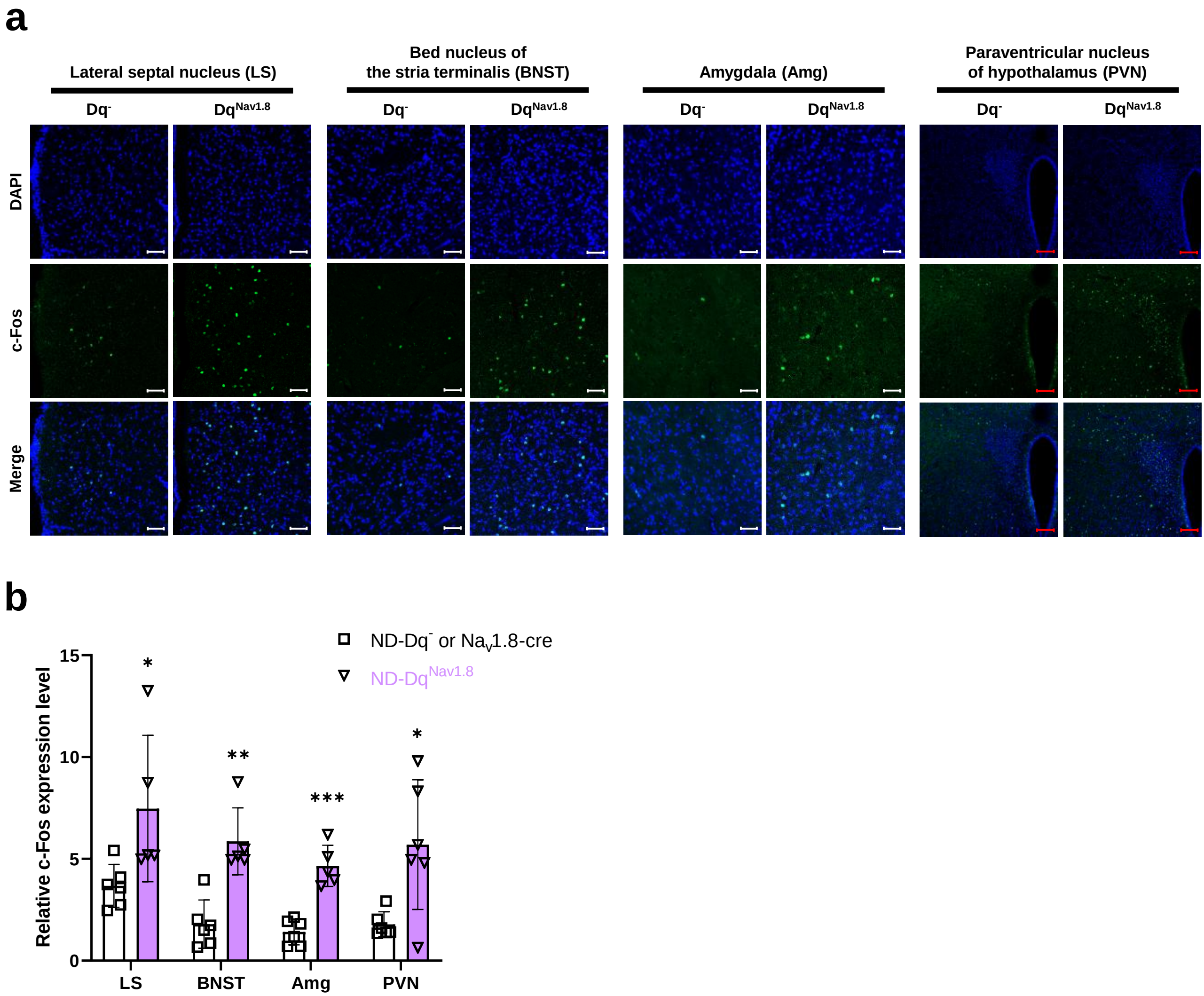

Supplementary Fig. 7. Intraperitoneal administration of CNO induces c-Fos immunoreactivity in Na$_v$1.8-expressing neurons. (a) Representative immunofluorescence images of mouse brain sections stained for c-Fos (green) and counterstained with DAPI (blue). White scale bar, 50 µm; red scale bar, 100 µm. (b) Quantification of c-Fos positive cells relative to DAPI-positive cells. Statistical analysis was conducted using an unpaired t-test. Data are presented as mean ± SEM (n=6 ND-Dq$^-$, n=6 ND-Na$_v$1.8-cre, and n=5-6 ND-Dq$^{Nav1.8}$); * *P* < .05. ** *P* < .01. *** *P* < .001. CNO, clozapine-N-oxide.

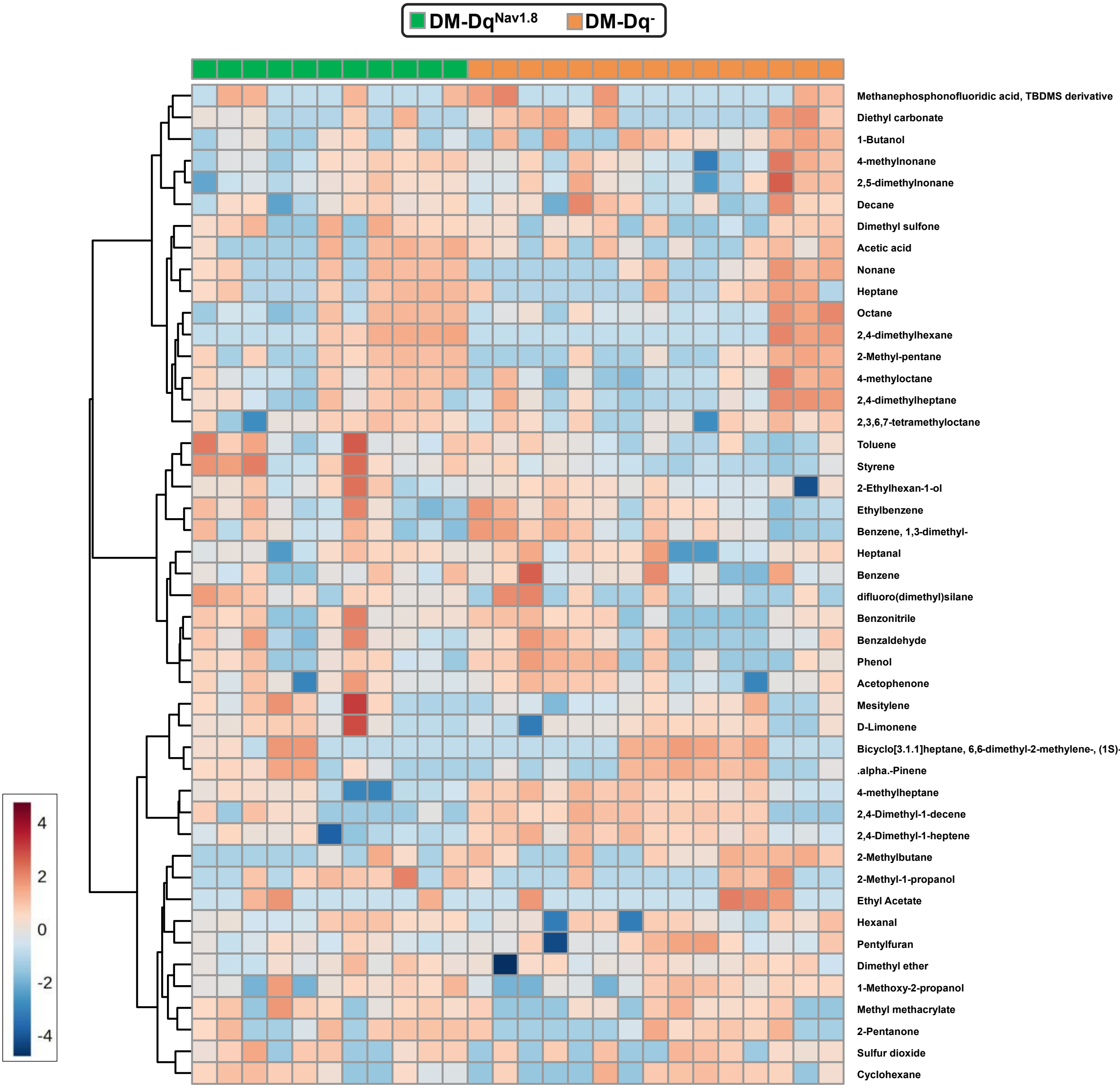

Supplementary Fig. 8. Contrasting patterns of breath VOCs in the DM-Dq$^-$ and DM-Dq$^{Nav1.8}$ mice after CNO injection. Hierarchical clustering heatmap of breath VOCs generated using Ward's clustering method (orange, DM-Dq$^-$; green: DM-Dq$^{Nav1.8}$).

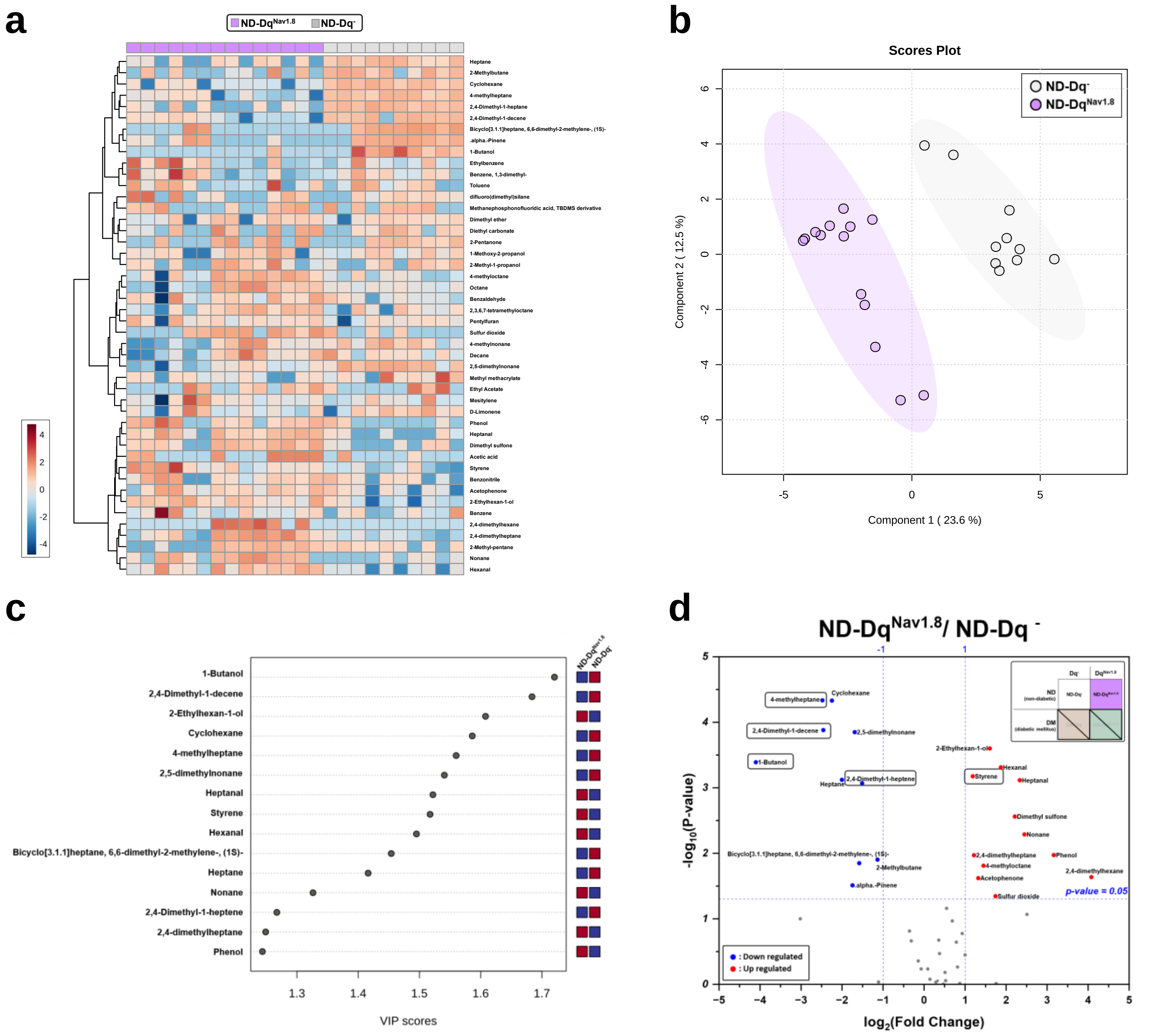

e

ND-Dq$^{Nav1.8}$ / ND-Dq^{-}					
**Name of volatile organic compounds**	**Log$_2$(Fold Change)**	**p-value**	**Name of volatile organic compounds**	**Log$_2$(Fold Change)**	**p-value**
**Cyclohexane**	-2.4773	0.0000464	**Dimethyl sulfone**	2.2162	0.002757
**4-methylheptane**	-2.2448	0.0000469	**Nonane**	2.4501	0.005163
**2,4-Dimethyl-1-decene**	-2.455	0.000132	**Phenol**	3.165	0.010694
**2,5-dimethylnonane**	-1.6889	0.000142	**2,4-dimethylheptane**	1.2196	0.010735
**2-Ethylhexan-1-ol**	1.6036	0.000252	**2-Methylbutane**	-1.1324	0.012561
**1-Butanol**	-4.0994	0.000409	**Bicyclo[3.1.1]heptane, 6,6-dimethyl-2-methylene-, (1S)-**	-1.579	0.014221
**Hexanal**	1.8752	0.000488	**4-methyloctane**	1.4521	0.015536
**Styrene**	1.1889	0.00067	**2,4-dimethylhexane**	4.0824	0.023167
**2,4-Dimethyl-1-heptane**	-2.0011	0.000758	**Acetophenone**	1.3239	0.024052
**Heptanal**	2.3351	0.00077	**.alpha.-Pinene**	-1.7434	0.030967
**Heptane**	-1.5089	0.000857	**Sulfur dioxide**	1.7426	0.045247

Supplementary Fig. 9. Contrasting patterns of breath VOCs in the ND-Dq^{-} and ND-Dq$^{Nav1.8}$ mice after CNO injection. (a) Hierarchical clustering heatmap of breath VOCs generated using Ward's clustering method (white, ND-Dq^{-}; purple, ND-Dq$^{Nav1.8}$). (b) PLS-DA plot visualizing grouping patterns of breath VOC concentrations in the ND-Dq$^{Nav1.8}$ and ND-Dq^{-} mice. (c) VIP plot displaying the top 15 breath VOCs identified with the PLS-DA method (box colors on the right of the chart indicate the intensity of each VOC; red, higher; blue, lower) (d) Volcano plot comparing breath VOCs between ND-Dq$^{Nav1.8}$ and ND-Dq^{-} mice after CNO injection, showing differential VOCs identified using the Wilcoxon rank-sum tests ($|\log_2 FC| \geq 1.0$, $P < .05$; ND-Dq$^{Nav1.8}$/ND-Dq^{-}). Rectangular boxes around VOC names in Supplementary Fig. 9d and Fig. 4c indicate commonly detected VOCs following activation of $Na_v1.8$-expressing neurons. (e) Table of breath VOCs identified as statistically meaningful ($|\log_2 FC| \geq 1.0$, $P < .05$) from a comparison of ND-Dq$^{Nav1.8}$ and ND-Dq^{-} mice.

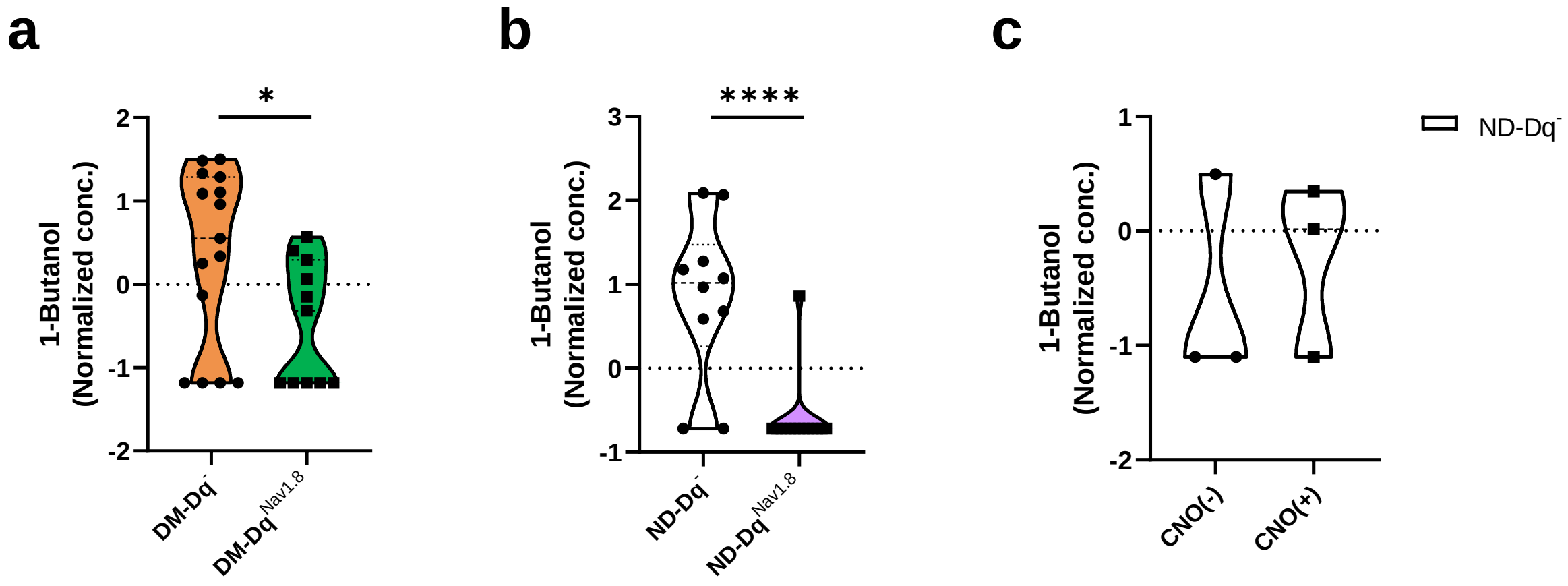

Supplementary Fig. 10. Normalized concentrations of 1-butanol in DM and ND mice depending on the activation of Na_v1.8-expressing neurons. Comparison of 1-butanol concentrations between (a) DM-Dq$^-$ and DM-Dq$^{Nav1.8}$ mice (n=15 DM-Dq$^-$ and n=11 DM-Dq$^{Nav1.8}$), and (b) ND-Dq$^-$ and ND-Dq$^{Nav1.8}$ mice (n=10 ND-Dq$^-$ and n=14 ND-Dq$^{Nav1.8}$). (c) Comparison of 1-butanol concentrations in ND-Dq$^-$ mice before (-) and after (+) CNO administration (n=3 CNO(-) and CNO(+) ND-Dq$^-$). Statistical analysis was conducted using an unpaired t-test. Data are presented as mean ± SEM. * P < .05. **** P < .0001.

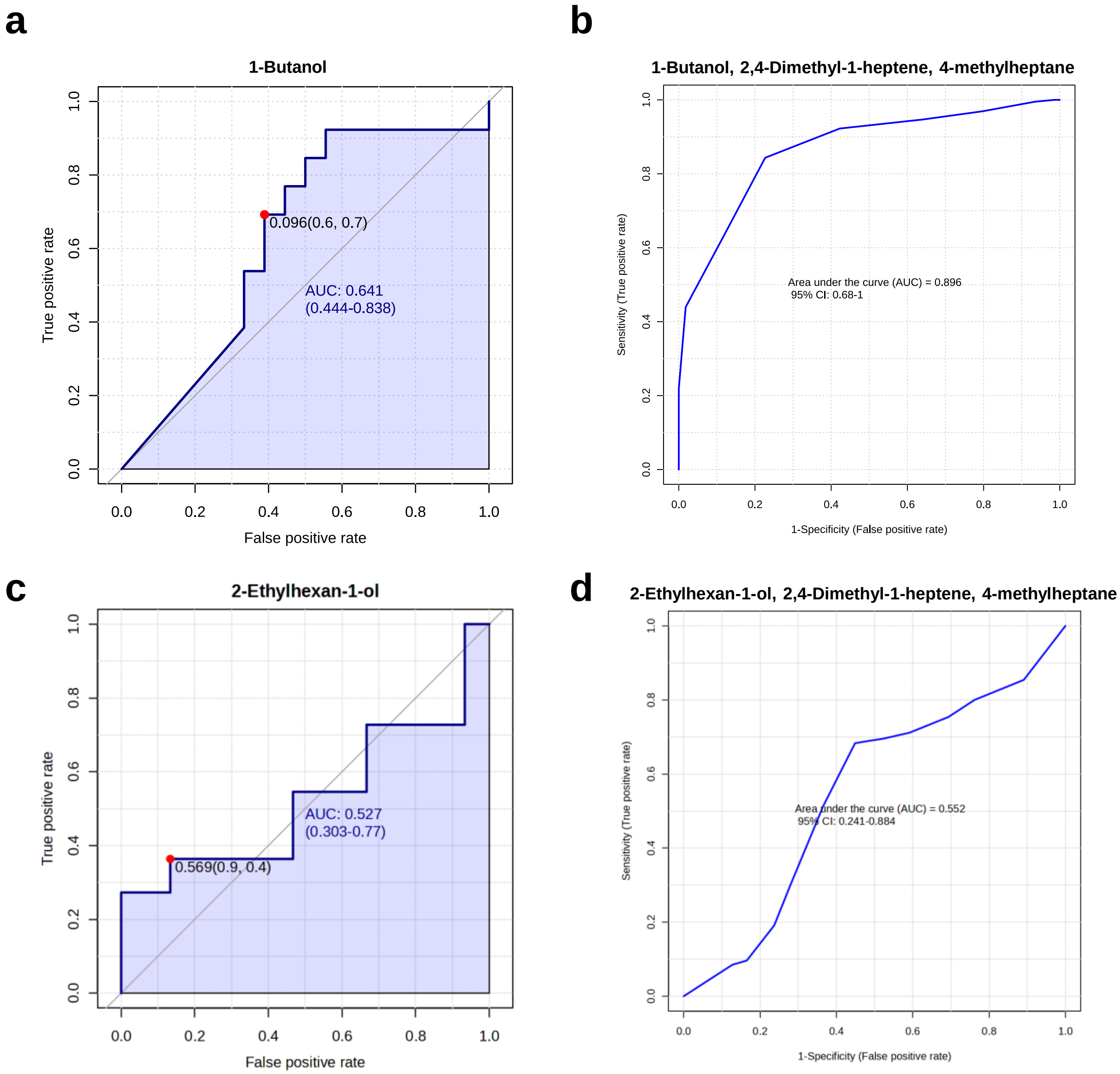

Supplementary Fig. 11. Evaluation of VOC-based test models for DG treatment response. (a) Receiver Operating Characteristic (ROC) curve to test a univariate model with 1-butanol. (b) ROC curve of a multivariate model with 1-butanol, 2,4-dimethyl-1-heptene and 4-methylheptane. (c) ROC curve to test a univariate model with 2-ethylhexan-1-ol. (d) ROC curve of a multivariate model with 2-ethylhexan-1-ol, 2,4-dimethyl-1-heptene and 4-methylheptane. CI, confidence interval.
